# Infrared pupillometry to help predict neurological outcome for patients achieving return of spontaneous circulation following cardiac arrest: a systematic review protocol

**DOI:** 10.1186/s13643-019-1209-z

**Published:** 2019-11-25

**Authors:** Alex Monk, Shashank Patil

**Affiliations:** 1 Emergency Department, Chelsea and Westminster Hospital, 369 Fulham Road, Chelsea, London, SW10 9NH England; 2 Kings College London, Room No 213, St Thomas’ House, Westminster Bridge Road, London, SE1 7EH England

**Keywords:** Cardiac arrest, Prognostication, Infrared pupillometry, Systematic review

## Abstract

**Background:**

Despite advances in resuscitation care, mortality rates following cardiac arrest (CA) remain high. Between one-quarter (in-hospital CA) and two-thirds (out of hospital CA) of patients admitted comatose to intensive care die of neurological injury. Neuroprognostication determines an informed and timely withdrawal of life sustaining treatment (WLST), sparing the patient unnecessary suffering, alleviating family distress and allowing a more utilitarian use of resources. The latest Resuscitation Council UK (2015) guidance on post-resuscitation care provides the current multi-modal neuroprognostication strategy to predict neurological outcome. Its modalities include neurological examination, neurophysiological tests, biomarkers and radiology. Despite each of the current strategy’s predictive modalities exhibiting limitations, meta-analyses show that three, namely PLR (pupillary light reflex), CR (corneal reflex) and N20 SSEP (somatosensory-evoked potential), accurately predict poor neurological outcome with low false positive rates. However, the quality of evidence is low, reducing confidence in the strategy’s results. While infrared pupillometry (IRP) is not currently used as a prognostication modality, it can provide a quantitative and objective measure of pupillary size and PLR, giving a definitive view of the second and third cranial nerve activity, a predictor of neurological outcome.

**Methods:**

The proposed study will test the hypothesis, “in those patients who remain comatose following return of spontaneous circulation (ROSC) after CA, IRP can be used early to help predict poor neurological outcome”. A comprehensive review of the evidence using a PRISMA-P (2015) compliant methodology will be underpinned by systematic searching of electronic databases and the two authors selecting and screening eligible studies using the Cochrane data extraction and assessment template. Randomised controlled trials and retrospective and prospective studies will be included, and the quality and strength of evidence will be assessed using the Grading  of Recommendation, Assessment and Evaluation (GRADE) approach.

**Discussion:**

IRP requires rudimentary skill and is objective and repeatable. As a clinical prognostication modality, it may be utilised early, when the strategy’s other modalities are not recommended. Corroboration in the evidence would promote early use of IRP and a reduction in ICU bed days.

**Systematic review registration:**

PROSPERO CRD42018118180

## Background

Despite advances in resuscitation care, mortality rates following cardiac arrest (CA) remain high [1, 2]. Between one-quarter (in-hospital CA) and two-thirds (out of hospital CA) of patients admitted comatose to intensive care, die of neurological injury [3], the majority as a result of withdrawal of life sustaining treatment (WLST) following a poor neuroprognosis [4]. While neuroprognosis determines an informed and timely WLST decision, it is complicated by a range of factors.

**Fig. 1:**
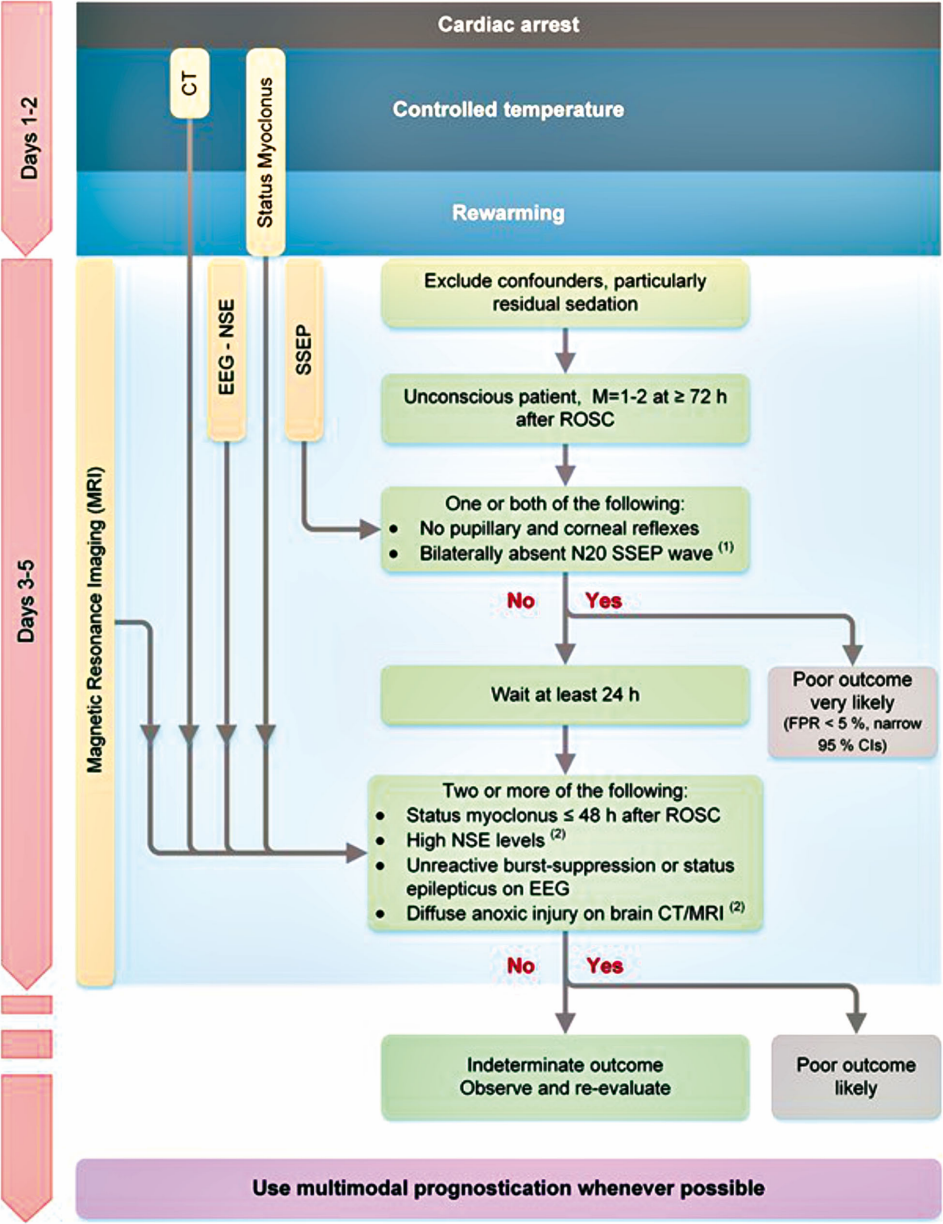
Prognostication strategy

- On return of spontaneous circulation (ROSC) following cardiac arrest, management of post-cardiac arrest syndrome [5] can be particularly challenging. Its components, brain injury, myocardial dysfunction, systemic ischaemia/reperfusion response and persistent precipitating pathology are inter-related, with observable benefits depending on optimisation of all.
- The latest Resuscitation Council UK (2015) guidance on post-resuscitation care [4] guards against post-ischaemic neuronal injury to maximise neurological recovery. The guidance includes adequate sedation and targeted temperature management (TTM), which can *reduce the accuracy of prognostic modalities* [6, 7].
- A systematic review of international data on prognostication modalities in comatose post-CA patients treated with TTM [8] gave rise to a multi-modal strategy for prognostication [9, 10], published jointly by ERC-ESICM (European Resuscitation Council and European Society of Intensive Care Medicine) [11, 12]. The strategy (Fig. 1) advises that prognostication is initially *delayed 72 h* after ROSC to allow rewarming and clearance of residual sedation [12].
- The prognostication strategy’s modalities are differentiated in the guidance by their specificity, precision and robustness. The more robust modalities:
  - bilaterally absent pupillary light reflex (PLR);
  - corneal reflex (CR) and
  - bilaterally absent N20 SSEP (somatosensory-evoked potential) wave;

are used first and their combined results used to prognosticate [6, 12]. When a poor neurological outcome is not predicted to be ‘very likely’, less robust modalities are added after a *further 24-h delay* [6, 12].

- Each predictive modality exhibits limitations. Studies show that some are subjective or prone to inconsistencies [13–16]. Others rely on specialist interpretation [17–20], are ill-defined [4] or are incompletely understood [6].
- Meta-analyses of the strategy’s modalities show that PLR, CR and N20 SSEP predict poor neurological outcome with low false positive rates [8, 21]. However, the primary studies provided ‘low’ to ‘very low’ quality of evidence [4, 6, 7, 21], reducing confidence in the prediction and therefore *extending the observation period*.
- The primary studies show that WLST is influenced by a self-fulfilling prophecy, a bias introduced when prognostic modalities are not blinded to the treating team [4, 6]. As well as reducing the quality of the evidence, this bias reduces available evidence on delayed awakening (late recovery of consciousness following coma), which can affect 30% of post-CA patients [22]^.^

The complex management of post-CA syndrome, neuroprotective measures’ adverse impact on prognostication accuracy (sedation and TTM), consequent delays in prognostication (72 h and 24 h), limitations of the individual modalities and unblinded observer bias, all present a challenge to early prediction of a poor neurological outcome.

The immediate consequence of a delayed poor neuroprognosis is futile treatment and resource wastage. Treatment is multifaceted, complex and costly. A 2015 study [23] (P.6) found ‘a significant correlation between length of stay and cost, with a much longer length of stay in ICU and hospital for CPC (Glasgow-Pittsburgh Cerebral Performance Category [24]) 3–4 patients’ (cf. CPC 1–2).

The intended benefit of treatment, quality adjusted life years (QALY), has a complex relationship with neurological status. Petrie et al showed that cost per QALY for ROSC post-CA, high-quality (CPC 1-2) survivors, at £16,000 [23], is well within the £30,000 UK (NICE) threshhold [25]. However, Petrie et al noted that ‘a major determinant of cost for the CPC 1–2 group was the burden of cost of the non-survivors and CPC 3–4 patient group [23] (p.6). An aim of the proposed review is earlier neuroprognosis in CPC 3–4 patients, which will reduce this cost burden.

In principle, improving the current multi-modal prognostication strategy to enable earlier prediction of poor neurological outcome would allow a more utilitarian resource allocation, reduce futile treatment and lessen the healthcare opportunity cost resource depletion imposes. The usefulness of supplementary modalities that yield immediate results, require rudimentary operator skill and are amenable to easily repeatable tests is being investigated [20].

IRP is emerging as one such promising prognostic modality, with one recent study showing that it yields higher specificity and sensitivity than manual PLR measurement [26]. In IRP, infrared light is shone directly at the pupil and data is obtained through analysis of the reflected image. Characteristics of the PLR include, amplitude, latency constriction and dilatation velocity. This provides a direct functional assessment of the second and third cranial nerves, a predictor of neurological outcome.

## Objectives

The proposed study will comprehensively review the evidence to determine whether the early use of IRP would help predict neurological outcome in comatose patients who achieve ROSC following CA. Questions of particular interest include the following:

- Timeliness - Can IRP be used early in the prognostication strategy to inform an earlier WLST decision?
- Specificity - Can IRP reduce the risk of falsely pessimistic prediction, reducing the lack of confidence that increases observation periods and inflates cost?
- Sensitivity - Can IRP reduce the incidence of delayed neuroprognoses, reducing ICU bed days?
- Primary hypothesis - In those patients who remain comatose following ROSC after CA, IRP can be used early to help predict poor neurological outcome.

## Methods

The design of the systematic review will follow the PRISMA-P 2015 checklist [27] (Additional file 1). The systematic review protocol is registered with PROSPERO under ID ‘CRD42018118180’ and is provided in Additional file 2.

### Eligibility criteria

This systematic review will consider randomised controlled trials, systematic reviews and retrospective and prospective cohort studies with specific study characteristics. Study populations will include adults over the age of 18 who suffered a cardiac arrest; the intervention will be infrared pupillometry performed early in the prognostication strategy; the primary outcome measure is neurological outcome.

Studies will be excluded if their study populations included cardiac arrests of traumatic aetiology, pregnant women or paediatric cases. Case reports will also be excluded.

Only published studies written in English language will be considered. Year of publication will not form part of the exclusion criteria.

### Information sources

A comprehensive and systematic search of the following electronic databases will inform this systematic review. The Healthcare Databases Advanced Search (HDAS), accessed through NICE, will be used as the interface through which, EMBASE, MEDLINE and CINAHL databases are searched. The Cochrane Database of Systematic Reviews (CDSR) will be searched. A search for completed systematic reviews within PROSPERO will also be carried out. The search will be expanded through direct contact with authors of works’ pending publication, reference mining and citation searching of the related literature and hand searching of relevant journals.

### Search strategy

The search strategy was defined and applied by a specialist search strategist (PB). HDAS has the advantage that a common syntax can be used to search EMBASE, MEDLINE and CINAHL, albeit using controlled vocabulary appropriate to each database. Natural language keyword searches include ‘cardiac arrest’, ‘prognosis’ and ‘infrared pupillometry’. Boolean logic was used to combine search terms and allow extraction of potentially suitable abstracts. The completed search strategy for EMBASE, MEDLINE and CINAHL is provided in Additional file 3; this will be peer reviewed, using the Peer Review of Electronic Search Strategies (PRESS [28]) checklist, by an independent information specialist.

### Data management

The extracted abstracts will be managed in HDAS.

### Study selection process and data collection process

The study selection process will follow the PRISMA 2009 flow diagram from the PRISMA statement [27]. Extracted abstracts will be screened for duplicates before review by two authors AM and SP. Abstracts that are not excluded during initial review will undergo full text screening by each author independently.

A modified version of the Cochrane data extraction and assessment template [29] (Additional file 4) will be used for full text screening and data extraction. Within this, any reasons for exclusion will be noted. Variable and outcome data will be extracted in the same template. When the required data is not specified in a study’s full text, all reasonable attempts will be made to contact the author and the content of any correspondence will be documented.

### Data items

The following variables will be extracted from eligible studies: patient age and gender, outcome of cardiac arrest and quantitative values of infrared pupillometry.

### Outcomes and prioritisation

The main outcome measure to be extracted from eligible studies is neurological outcome. Additional outcomes include the number of ICU bed days and survival at discharge.

### Risk of bias in individual studies

Risk-of-bias assessments will be made independently by two authors AM and SP. For all RCTs, we will use the Cochrane risk-of-bias (RoB) tool [30] and for all non-randomised studies, the ROBINS-1 tool [31].

The quality and strength of evidence will be assessed using the Grades of Recommendation, Assessment and Evaluation (GRADE) approach [32].

### Internal validity

Inter-reviewer agreement in study inclusion, data extraction and risk-of-bias assessments will be maximised by piloting the data extraction form and risk-of-bias tools prior to use in the systematic review. Clear usage instructions will include guidance on consistency of input styles and documentation of missing information [29].

Inter-reviewer agreement will be measured using the kappa statistic for the initial study screening, data extraction and risk-of-bias assessment.

A third reviewer will independently review the studies that elicit disagreement between AM and SP in study inclusion, data extraction or risk-of-bias assessments. Disagreements will be discussed between all three reviewers at regular team meetings. Each disagreement will be interrogated with reference to the decision rules and guidance on the use of the data extraction tool and risk-of-bias assessments. The outcome and reason for the initial disagreement will be recorded.

### Synthesis and meta-biases

This review aims to establish the association between IRP values that indicate a ‘very likely’ poor prognosis and patients with poor neurological outcome (defined as CPC 3–5).

IRP data will be correlated with patient outcome (Table 1) and employed in odds ratios to determine IRP’s effect as an additional modality within the neuroprognostic algorithm. Taking account of the results from all studies, we will calculate aggregated estimates of the effect of intervention, together with *p* values, means, confidence intervals and value ranges.

**Table 1:** Patient outcomes

	Poor outcome	Good outcome
‘Very likely’ poor prognosis	PP WLST	PG False positive
No ‘very likely’ poor prognosis	GP False negative	GG £16 k/QALY Survivors

A Forest plot will be used to graphically represent the size of the effect seen in individual studies and the summarised effect.

Of particular interest are IRP’s specificity, PP/(PP + PG); sensitivity, PP/(PP + GP); false positive rate, PG/(PP + PG) and its false negative rate, GP/(GP + GG).

Variation will be checked for between studies (heterogeneity) using Cochran’s Q and other I-square statistics [33]. If significant heterogeneity is found, we will apply the random-effects model [34]. To identify sources of heterogeneity and adjust for them, sub-group analysis will be performed and meta-regression will be used to identify the influence co-variates have on the overall effect.

Homogeneity will be addressed using the fixed effects model [34].

### Confidence in cumulative evidence

The quality and strength of evidence will be assessed using the Grades of Recommendation, Assessment and Evaluation (GRADE) approach [32].

## Discussion

The current multi-modal neuroprognostication strategy advises that following ROSC after CA, clinicians wait 72 h to allow rewarming and clearance of sedation before prognosticating. A poor neuroprognosis prompts WLST, any usable strategy must minimise the risk of falsely pessimistic predictions, the false positive rate.

Despite each of the current strategy’s predictive modalities exhibiting limitations, meta-analyses of the modalities show that PLR, CR and N20 SSEP predict poor neurological outcome with low false positive rates [8, 21]. However, the quality of evidence is low, thereby reducing confidence in the strategy’s results.

In the clinical utilisation of the strategy, false positive risk is mitigated by extending observation periods and utilising additional modalities [6]. Concomitantly, risk aversion increases the false negative rate (lack of a poor prognosis preceding a poor neurological outcome). The costs of risk mitigation [23] can be reduced through earlier prognostication using greater-specificity/greater-sensitivity modalities that are objective, repeatable and can be deployed early in the strategy using readily available expertise. Greater specificity will increase confidence in predictions of poor neurological outcome, obviating the need to extend the observation period, and greater sensitivity will reduce the false negative rate.

IRP’s characteristics, objectivity, repeatability and rudimentary operation make it a promising candidate for review. The proposed study will comprehensively review the evidence for IRP and determine whether it will help predict a poor neurological outcome post-CA.

## Supplementary information

**Additional file 1.** PRISMA-P (Preferred Reporting Items for Systematic review and Meta-Analysis Protocols) 2015 checklist: recommended items to address in a systematic review protocol.

**Additional file 2.** PROSPERO registration document.

**Additional file 3.** Search Strategy on EMBASE, MEDLINE and CINAHL.

**Additional file 4.** Cochrane public health group data extraction and assessment template.

## Data Availability

All data pertaining to this review protocol can be found within the body of text or in the supplementary additional files.

## Abbreviations

AM: Alex Monk
CA: Cardiac arrest
CDSR: Cochrane Database of Systematic Reviews
CPC: Glasgow-Pittsburgh Cerebral Performance Category
CR: Corneal reflex
CT: Computerised tomography
EEG: Electroencephalography
ERC: European Resuscitation Council
ESICM: European Society of Intensive Care Medicine
FPR: False positive rate
GG: No poor neuroprognosis—good neurological outcome
GP: No poor neuroprognosis—poor neurological outcome
GRADE: Grades of Recommendation, Assessment and Evaluation
HDAS: Healthcare Databases Advanced Search
ICU: Intensive care unit
IRP: Infrared pupillometry
MeSH: Medical Subject Headings
MRI: Magnetic resonance imaging
NICE: National Institute for Health and Care Excellence
NSE: Neuron-specific enolase
PB: Phillip Barlow
PG: Poor neuroprognosis—good neurological outcome
PLR: Pupillary light reflex
PP: Poor neuroprognosis—poor neurological outcome
PRESS: Peer Review of Electronic Search Strategies
PRISMA-P: Preferred reporting items for systematic review and meta-analysis protocols
QALY: Quality adjusted life year
RCT: randomised controlled trial
RoB: Risk of bias
Robins-I: Risk of bias in non-randomised studies of interventions
ROSC: Return of spontaneous circulation
SP: Shashank Patil
SSEP: Somatosensory-evoked potential
TTM: Targeted temperature management
WLST: Withdrawal of life sustaining treatment
