# Supplementary material for: Infrared pupillometry to help predict neurological outcome for patients achieving return of spontaneous circulation following cardiac arrest: a systematic review protocol
Source: Syst Rev. 2019 Nov 25;8:286. doi: 10.1186/s13643-019-1209-z (PMC6876106; doi:10.1186/s13643-019-1209-z)
Supplement: Supplementary file 2 — Additional file 2. PROSPERO registration document. [file 13643_2019_1209_MOESM2_ESM.pdf]

Infrared pupillometry to help predict neurological outcome for patients achieving return of spontaneous circulation (ROSC) following cardiac arrest: systematic review

Alex Monk, Shashank Patill, Lisa Ramage, Leanne Eveson

Citation

Alex Monk, Shashank Patill, Lisa Ramage, Leanne Eveson. Infrared pupillometry to help predict neurological outcome for patients achieving return of spontaneous circulation (ROSC) following cardiac arrest: systematic review. PROSPERO 2018 CRD42018118180 Available from: http://www.crd.york.ac.uk/PROSPERO/display_record.php?ID=CRD42018118180

Review question

Can infrared pupillometry help predict neurological outcome in patients who achieve return of spontaneous circulation (ROSC) following cardiac arrest.

Searches

We will search the following electronic bibliographic databases: MEDLINE, EMBASE, CINAHL and The Cochrane Library (Cochrane Database of Systematic Reviews, Cochrane Central Register of Controlled Trials (CENTRAL), Cochrane Methodology Register).

Types of study to be included

Retrospective studies, Prospective cohort studies, Randomised controlled trials, Systematic reviews.

Condition or domain being studied

Neurological outcome in patients achieving return of spontaneous circulation following cardiac arrest.

Participants/population

Adults over 18 years of age.

Intervention(s), exposure(s)

Inclusion criteria:

Cardiac arrest in adults over 18 years of age

Use of infrared pupillometry performed within 24 hours of ROSC

Neurological outcome

Exclusion criteria:

Case reports

Paediatric studies

Studies including pregnant women

Non English studies

Cardiac arrest of non cardiac aeitiology.

Comparator(s)/control

Patients who had no such intervention or use of the current prognostication guidance will form the control group.

Context

Main outcome(s)
Ability to predict neurological outcome using infrared pupillometry as a quantitative tool.

Timing and effect measures

Within 24 hours of presentation to hospital: Emergency department or ITU

The impact of targeted temperature management on ICU stays.

Additional outcome(s)
Prediction of survival.

Prediction of ROSC.

Data extraction (selection and coding)
- A search strategist will run searches on above mentioned databases using key words.

- 2 reviewers will independently review the abstracts, include all the papers agreed on.

- If different views on certain papers, an opinion will be sought from a third reviewer

Data to be extracted:

- Outcome of cardiac arrest

- Neurological outcome

- Survival at discharge

- Number of days on ITU

- Quantitative value of infrared pupillometry.

Risk of bias (quality) assessment
For RCT we will use the Cochrane ROB tool.

For non RCTs - ROBINS-I tool.

Strategy for data synthesis
We will use Grades of Recommendation, Assessment, Development and Evaluation (GRADE) system for analysis of selected studies.

Results will be stored in HDAS (Healthcare Database Advanced Search)

We will use Cochrane template for data extraction.

Analysis of subgroups or subsets
None at this point, will be reviewed if need be.

Contact details for further information
Dr Shashank Patil
shashank.patil@chelwest.nhs.uk

Organisational affiliation of the review
Chelsea and Westminster NHS Foundation Trust
http://www.chelwest.nhs.uk/

Review team members and their organisational affiliations
Dr Alex Monk. Chelsea and Westminster NHS Foundation Trust

Dr Shashank Patill. Chelsea and Westminster NHS Foundation Trust
Dr Lisa Ramage. Chelsea and Westminster NHS Foundation Trust
Dr Leanne Eveson. Chelsea and Westminster NHS Foundation Trust

Anticipated or actual start date
01 January 2019

Anticipated completion date
30 June 2019

Funding sources/sponsors
None.

Conflicts of interest

Language
English

Country
England

Stage of review
Review_Ongoing

Subject index terms status
Subject indexing assigned by CRD

Subject index terms
Cardiopulmonary Resuscitation; Heart Arrest; Humans

Date of registration in PROSPERO
13 December 2018

Date of publication of this version
13 December 2018

Details of any existing review of the same topic by the same authors

Stage of review at time of this submission

Stage	Started	Completed
Preliminary searches	Yes	Yes
Piloting of the study selection process	No	No
Formal screening of search results against eligibility criteria	No	No
Data extraction	No	No
Risk of bias (quality) assessment	No	No
Data analysis	No	No

Versions
13 December 2018

faith and registered the review in PROSPERO. CRD bears no responsibility or liability for the content of this registration record, any associated files or external websites.
