# Supplementary material for: Infrared pupillometry to help predict neurological outcome for patients achieving return of spontaneous circulation following cardiac arrest: a systematic review protocol
Source: Syst Rev. 2019 Nov 25;8:286. doi: 10.1186/s13643-019-1209-z (PMC6876106; doi:10.1186/s13643-019-1209-z)
Supplement: Supplementary file 3 — Additional file 3. Search Strategy on EMBASE, MEDLINE and CINAHL. [file 13643_2019_1209_MOESM3_ESM.pdf]

Strategy 406474/saved

See full search strategy

Contents 62 of 62 results on Saved Results

1. Early prediction of coma recovery after cardiac arrest with blinded pupillometry. .... Page 4

2. Neuroprognostication after cardiac arrest in the light of targeted temperature management........ Page 4

3. Quantitative pupillometry and transcranial Doppler measurements in patients treated with hypothermia after cardiac arrest. .... Page 4

4. Does this comatose survivor of cardiac arrest have a poor prognosis?........ Page 5

5. Automated quantitative pupillometry for the prognostication of coma after cardiac arrest. .... Page 5

6. Paralytics Use Should be Cautioned in the Prediction for Prognosis After Hypoxic Injury...Pediatr Crit Care Med. 2012 Jan;13(1):32-8 .... Page 6

7. Extracorporeal Cardiopulmonary Resuscitation for Patients With Out-of-Hospital Cardiac Arrest of Cardiac Origin: A Propensity-Matched Study and Predictor Analysis*........ Page 6

8. Outcome prediction by motor and pupillary responses in children treated with therapeutic hypothermia after cardiac arrest. .... Page 6

9. Looking into the crystal ball: Can we predict prognosis in children treated with therapeutic hypothermia after cardiac arrest?*........ Page 7

10. Reversible brain death after cardiopulmonary arrest and induced hypothermia. .... Page 7

11. Multicenter cohort study of out-of-hospital pediatric cardiac arrest. .... Page 8

12. Predictors of neurologic outcome in hypothermia after cardiac arrest. .... Page 8

13. Association between clinical examination and outcome after cardiac arrest. .... Page 9

14. Predictors of poor neurologic outcome after induced mild hypothermia following cardiac arrest...Neurology. 2008 Nov 4;71(19):1535-7 .... Page 9

15. A clinical and EEG scoring system that predicts early cortical response (N20) to somatosensory evoked potentials and outcome after cardiac arrest. .... Page 9

16. Survival from cardiac arrest in the Accident and Emergency Department........ Page 10

17. Clinical reasoning: Prognostication after cardiac arrest: What do we really know?........ Page 10

18. Prognostic impact of coagulopathy at hospital admission on 30-day neurological outcomes in patients with out-of-hospital cardiac arrest........ Page 10

19. Multimodal Outcome Prognostication After Cardiac Arrest and Targeted Temperature Management: Analysis at 36 degreeC........ Page 11

20. Predictors of functional outcome after hanging injury .... Page 11

21. Role of electrophysiologic studies in the first 72 hours in comatose postcardiac arrest patients........ Page 12

22. Very early pupillometry, eeg suppression, and bis data predict outcome 6 hours after cardiac arrest .... Page 12

23. Cerebral vasomotor reactivity test during targeted temperature management is feasible for prognostication after out of hospital cardiac arrest........ Page 13

24. Automated pupillometry for early prognostication in comatose cardiac arrest patients: Preliminary results of a multicenter study........ Page 13

25. Neurological pupil index predicts neurological outcome early after cardiac arrest: An observational study .... Page 14

26. Post-anoxic quantitative MRI changes may predict emergence from coma and functional outcomes at discharge .... Page 14

27. Pain-related Somato Sensory Evoked Potentials: A potential new tool to improve the prognostic prediction of coma after cardiac arrest ..... Page 15

28. Early Absent Pupillary Light Reflexes after Cardiac Arrest in Patients Treated with Therapeutic Hypothermia..... Page 15

29. Neuron specific enolase and Glasgow motor score remain useful tools for assessing neurological prognosis after out-of-hospital cardiac arrest treated with therapeutic hypothermia ..... Page 16

30. Prospective Cohort Study Evaluating the Prognostic Value of Simple EEG Parameters in Postanoxic Coma ..... Page 16

31. Clinical and EEG characteristics of patients with preserved cortical SSEPS after post-anoxic coma ..... Page 17

32. The clinical characteristics of patients with preserved cortical evoked potentials after post-anoxic coma and poor neurological outcome ..... Page 17

33. Neurological prognostication after cardiac arrest and targeted temperature management 33degreeC versus 36degreeC: Results from a randomised controlled clinical trial ..... Page 18

34. Amplitudes of SSEP and outcome in cardiac arrest survivors ..... Page 18

35. Target temperature management of 33degreeC and 36degreeC in patients with out-of-hospital cardiac arrest with initial non-shockable rhythm - A TTM sub-study ..... Page 19

36. Outcomes of asphyxial cardiac arrest patients who were treated with therapeutic hypothermia: A multicentre retrospective cohort study..... Page 19

37. The characteristics of patients with bilateral absent evoked potentials after post-anoxic injury ..... Page 20

38. Yeast central nervous system infection in a critically ill patient: A case report..... Page 20

39. Clinical examination for prognostication in comatose cardiac arrest patients ..... Page 21

40. Moderate therapeutic hypothermia in a patient with acute pancreatitis: Case report and review of the literature ..... Page 21

41. Self-fulfilling prophecies through withdrawal of care: Do they exist in traumatic brain injury, too? ..... Page 21

42. Usefulness of a simple prognostication score in prediction of the prognoses of patients with out-of-hospital cardiac arrests..... Page 22

43. Infrared pupillometry to detect the light reflex during cardiopulmonary resuscitation: A case series ..... Page 22

44. A case of inaccurate prognostication after the ARCTIC protocol..... Page 23

45. Utility cranial nerve testing obtained in the emergency department as early predictors of neurologic outcome post cardiac arrest ..... Page 23

46. Predictors of poor neurologic outcome in patients after cardiac arrest treated with hypothermia: A retrospective study ..... Page 23

47. Atropine, fixed dilated pupils and prognostication following cardiac arrest ..... Page 24

48. Sedation confounds outcome prediction in cardiac arrest survivors treated with hypothermia ..... Page 24

49. Prognostic value of brain diffusion- after weighted imaging cardiac arrest ..... Page 24

50. Neurologic prognosis after cardiac arrest ..... Page 25

51. Prognosis and prognostication after cardiac arrest and hypothermia; Results of PROPACII,, a Dutch multicenter, prospective cohort study ..... Page 25

52. Coma anoxico-isquemico en 46 pacientes. Evolucion a treinta dias y su relacion con los reflejos de troncoThe absence of brainstem reflexes does not predict short term mortality in anoxic ischemic coma..... Page 26

53. Coma in the intensive care unit: Predicting awakening following cardiac and respiratory arrest..... Page 26

54. Anoxic-ischemic encephalopathy: Clinical and electrophysiological associations with outcome ..... Page 27

55. Early prognostic criteria for neurological recovery and long-term survival after cardiac arrest..... Page 27

56. Cardiopulmonary resuscitation: A retrospective review ..... Page 28
57. [Multi-recurrent cardiac arrest: when to discontinue cardiopulmonary resuscitation?]. ..... Page 28
58. Predictors for good cerebral performance among adult survivors of out-of-hospital cardiac arrest. ..... Page 28
59. Physiologic predictors of survival in post-traumatic arrest. ..... Page 29
60. Survey of brain temperature management in patients with traumatic brain injury in the Japan neurotrauma data bank. ..... Page 29
61. Neurologic prognosis after cardiopulmonary arrest: IV. Brainstem reflexes. ..... Page 30
62. Prediction of awakening after out-of-hospital cardiac arrest. ..... Page 30
Full search strategy ..... Page 31

Results Saved Results
62 of 62 saved results

1. Early prediction of coma recovery after cardiac arrest with blinded pupillometry.

Authors Solari, Daria; Rossetti, Andrea O.; Carteron, Laurent; Miroz, John-Paul; Novy, Jan; Eckert, Philippe; Oddo, Mauro
Source Annals of Neurology; Jun 2017; vol. 81 (no. 6); p. 804-810
Publication Date Jun 2017
Publication Type(s) Academic Journal
PubMedID 28470675
Database CINAHL
Available at Annals of Neurology from Wiley Online Library Science , Technology and Medicine Collection 2017
Abstract Objective: Prognostication studies on comatose cardiac arrest (CA) patients are limited by lack of blinding, potentially causing overestimation of outcome predictors and self-fulfilling prophecy. Using a blinded approach, we analyzed the value of quantitative automated pupillometry to predict neurological recovery after CA.Methods: We examined a prospective cohort of 103 comatose adult patients who were unconscious 48 hours after CA and underwent repeated measurements of quantitative pupillary light reflex (PLR) using the Neurolight-Algiscan device. Clinical examination, electroencephalography (EEG), somatosensory evoked potentials (SSEP), and serum neuron-specific enolase were performed in parallel, as part of standard multimodal assessment. Automated pupillometry results were blinded to clinicians involved in patient care. Cerebral Performance Categories (CPC) at 1 year was the outcome endpoint.Results: Survivors (n = 50 patients; 32 CPC 1, 16 CPC 2, 2 CPC 3) had higher quantitative PLR (median = 20 [range = 13-41] vs 11 [0-55] %, p < 0.0001) and constriction velocity (1.46 [0.85-4.63] vs 0.94 [0.16-4.97] mm/s, p < 0.0001) than nonsurvivors. At 48 hours, a quantitative PLR < 13% had 100% specificity and positive predictive value to predict poor recovery (0% false-positive rate), and provided equal performance to that of EEG and SSEP. Reduced quantitative PLR correlated with higher serum neuron-specific enolase (Spearman r = -0.52, p < 0.0001).Interpretation: Reduced quantitative PLR correlates with postanoxic brain injury and, when compared to standard multimodal assessment, is highly accurate in predicting long-term prognosis after CA. This is the first prognostication study to show the value of automated pupillometry using a blinded approach to minimize self-fulfilling prophecy. Ann Neurol 2017;81:804-810.

2. Neuroprognostication after cardiac arrest in the light of targeted temperature management.

Authors Oddo, Mauro; Friberg, Hans
Source Current Opinion in Critical Care; Jun 2017; vol. 23 (no. 3); p. 244-250
Publication Date Jun 2017
Publication Type(s) Academic Journal
PubMedID 28323720
Database CINAHL
Available at Current Opinion in Critical Care from Ovid (Journals @ Ovid) - Remote Access
Available at Current Opinion in Critical Care from Ovid (LWW Total Access Collection 2015 - Q1 with Neurology)
Abstract Purpose Of Review: Delayed awakening after targeted temperature management (TTM) and sedation is frequent among cardiac arrest patients. Differentiating between prolonged coma and irreversible cerebral damage can be challenging, therefore the utilization of a multimodal approach is recommended by international guidelines. Here, we discuss indications and advantages/disadvantages of available modalities for coma prognostication and describe new tools to improve our accuracy for outcome prediction.Recent Findings: Studies from the TTM era confirmed that combining neurological examination with electrophysiological assessment [electroencephalography (EEG) and somato-sensory evoked potentials (SSEP)] greatly improves coma prognostication. This combination is nowadays recognized as the most useful by many clinicians and appears widely applicable as part of initial patient assessment. Additional tests (serum neuron specific enolase and neuroimaging) may be most useful to orient clinical decisions in patients with prolonged coma. Advanced analysis of EEG and SSEP recordings and the emergence of quantitative pupillometry hold great promise.Summary: Multimodal prognostication offers a comprehensive approach of anoxic-ischemic encephalopathy and is increasingly used in postresuscitation care. Worldwide implementation and future advancements of available modalities, together with the increasing use of novel automated devices for quantitative neurological examination, may further optimize prognostic accuracy in the early ICU phase following cardiac arrest.

3. Quantitative pupillometry and transcranial Doppler measurements in patients treated with hypothermia after cardiac arrest.

Authors Heimburger, Delphine; Durand, Michel; Gaide-Chevronnay, Lucie; Dessertaine, Geraldine; Moury, Pierre-Henri; Bouzat, Pierre; Albaladejo, Pierre; Payen, Jean-Francois
Source Resuscitation; Jun 2016; vol. 103 ; p. 88-93

Publication Date Jun 2016
Publication Type(s) Academic Journal
PubMedID 26970030
Database CINAHL
Abstract Background: Predicting outcome after cardiac arrest (CA) is particularly difficult when therapeutic hypothermia (TH) is used. We investigated the performance of quantitative pupillometry and transcranial Doppler (TCD) in this context.Methods: This prospective observational study included 82 post-CA patients. Quantitative assessment of pupillary light reflex (PLR) and TCD measurements of the two middle cerebral arteries were performed at admission (day 1) and after 24h (day 2) during TH (33-35°C) and sedation. Neurological outcome was assessed at 3 months using cerebral performance category (CPC) scores; patients were classified as having good (CPC 1-2) or poor (CPC 3-5) outcome. Prognostic performance was analyzed using area under the receiver operating characteristic curve (AUC-ROC).Results: Patients with good outcome (n=27) had higher PLR amplitude than patients with poor outcome (n=55) both at day 1, 13% (10-18) (median, 25th-75th percentile) vs. 8% (2-11) (P<0.001), and at day 2, 17% (13-20) vs. 8% (5-13) (P<0.001), respectively. The AUC-ROC curves at days 1 and 2 were 0.76 (95% confidence interval [CI] 0.65-0.86) and 0.82 (95% CI 0.73-0.92), respectively. The best cut-off values of PLR amplitude to predict a 3-month poor outcome were <9% and <11%, respectively. A PLR amplitude of <7% at day 2 predicted a 3-month poor outcome with a specificity of 100% (95% CI 86-100) and a sensitivity of 42% (95% CI 28-58). No differences in TCD measurements were found between the two patient groups.Conclusion: PLR measurements might be informative in the prediction of outcome of post-CA patients even under sedation and hypothermia.

4. Does this comatose survivor of cardiac arrest have a poor prognosis?

Authors Sandroni, Claudio; Soar, Jasmeet; Friberg, Hans
Source Intensive Care Medicine; Jan 2016; vol. 42 (no. 1); p. 104-106
Publication Date Jan 2016
Publication Type(s) Academic Journal
PubMedID 26527197
Database CINAHL
Available at Intensive Care Medicine from SpringerLink
Available at Intensive Care Medicine from ProQuest (Hospital Premium Collection) - NHS Version
Available at Intensive Care Medicine from EBSCOhost EJS
Abstract The article describes the case of a 62 year old man resuscitated from a witnessed ventricular fibrillation cardiac arrest preceded by acute chest pain. He was unconscious upon admission with his trachea intubated, lungs ventilated with unreactive pupils and no motor response. It suggests a multimodal approach based on clinical examination and diagnostic tests for reliable prognostication to prevent incorrect withdrawal of life-sustaining treatment (WLST) for presumed irreversible brain injury.

5. Automated quantitative pupillometry for the prognostication of coma after cardiac arrest.

Authors Suys, Tamarah; Bouzat, Pierre; Marques-Vidal, Pedro; Sala, Nathalie; Payen, Jean-François; Rossetti, Andrea O; Oddo, Mauro
Source Neurocritical Care; Oct 2014; vol. 21 (no. 2); p. 300-308
Publication Date Oct 2014
Publication Type(s) Academic Journal
PubMedID 24760270
Database CINAHL
Available at Neurocritical Care from SpringerLink
Abstract Background: Sedation and therapeutic hypothermia (TH) delay neurological responses and might reduce the accuracy of clinical examination to predict outcome after cardiac arrest (CA). We examined the accuracy of quantitative pupillary light reactivity (PLR), using an automated infrared pupillometry, to predict outcome of post-CA coma in comparison to standard PLR, EEG, and somato-sensory evoked potentials (SSEP).Methods: We prospectively studied over a 1-year period (June 2012-June 2013) 50 consecutive comatose CA patients treated with TH (33 °C, 24 h). Quantitative PLR (expressed as the % of pupillary response to a calibrated light stimulus) and standard PLR were measured at day 1 (TH and sedation; on average 16 h after CA) and day 2 (normothermia, off sedation: on average 46 h after CA). Neurological outcome was assessed at 90 days with Cerebral Performance Categories (CPC), dichotomized as good (CPC 1-2) versus poor (CPC 3-5). Predictive performance was analyzed using area under the ROC curves (AUC).Results: Patients with good outcome [n = 23 (46 %)] had higher quantitative PLR than those with poor outcome [n = 27; 16 (range 9-23) vs. 10 (1-30) % at day 1, and 20 (13-39) vs. 11 (1-55) % at day 2, both p < 0.001]. Best cut-off for outcome prediction of quantitative PLR was <13 %. The AUC to predict poor outcome was higher for quantitative than for standard PLR at both time points (day 1, 0.79 vs. 0.56, p = 0.005; day 2, 0.81 vs. 0.64, p = 0.006). Prognostic accuracy of quantitative PLR was comparable to that of EEG and SSEP (0.81 vs. 0.80 and 0.73, respectively, both p > 0.20).Conclusions: Quantitative PLR is more accurate than standard PLR in predicting outcome of post-anoxic coma, irrespective of temperature and sedation, and has comparable prognostic accuracy than EEG and SSEP.

6. Paralytics Use Should be Cautioned in the Prediction for Prognosis After Hypoxic Injury...Pediatr Crit Care Med. 2012 Jan;13(1):32-8

**Authors**	Chen, Yi-Chen; Yen, Ting-An; Wang, Ching-Chia
**Source**	Pediatric Critical Care Medicine; Jun 2013; vol. 14 (no. 5); p. 557-557
**Publication Date**	Jun 2013
**Publication Type(s)**	Academic Journal
**PubMedID**	23867441
**Database**	CINAHL

7. Extracorporeal Cardiopulmonary Resuscitation for Patients With Out-of-Hospital Cardiac Arrest of Cardiac Origin: A Propensity-Matched Study and Predictor Analysis*.

**Authors**	Maekawa, Kunihiko; Tanno, Katsutoshi; Hase, Mamoru; Mori, Kazuhisa; Asai, Yasufumi
**Source**	Critical Care Medicine; May 2013; vol. 41 (no. 5); p. 1186-1196
**Publication Date**	May 2013
**Publication Type(s)**	Academic Journal
**PubMedID**	23388518
**Database**	CINAHL<br>Available at Critical care medicine from Ovid (LWW Total Access Collection 2015 - Q1 with Neurology)<br>Available at Critical care medicine from Ovid (Journals @ Ovid) - London Health Libraries
**Abstract**	OBJECTIVE: : Encouraging results of extracorporeal cardiopulmonary resuscitation for patients with refractory cardiac arrest have been shown. However, the independent impact on the neurologic outcome remains unknown in the out-of-hospital population. Our objective was to compare the neurologic outcome following extracorporeal cardiopulmonary resuscitation and conventional cardiopulmonary resuscitation and determine potential predictors that can identify candidates for extracorporeal cardiopulmonary resuscitation among patients with out-of-hospital cardiac arrest of cardiac origin. DESIGN: : Post hoc analysis of data from a prospective observational cohort. SETTING: : A tertiary care university hospital in Sapporo, Japan (January 2000 to September 2004). PATIENTS: : A total of 162 adult patients with witnessed cardiac arrest of cardiac origin who had undergone cardiopulmonary resuscitation for longer than 20 minutes (53 in the extracorporeal cardiopulmonary resuscitation group and 109 in the conventional cardiopulmonary resuscitation group). INTERVENTIONS: : None. MEASUREMENTS AND MAIN RESULTS: : The primary endpoint was neurologically intact survival at three months after cardiac arrest. We used propensity score matching to reduce selection bias and balance the baseline characteristics and clinical variables that could potentially affect outcome. This matching process selected 24 patients from each group. The impact of extracorporeal cardiopulmonary resuscitation was estimated in matched patients. Intact survival rate was higher in the matched extracorporeal cardiopulmonary resuscitation group than in the matched conventional cardiopulmonary resuscitation group (29.2% [7/24] vs. 8.3% [2/24], log-rank p = 0.018). According to the predictor analysis, only pupil diameter on hospital arrival was associated with neurologic outcome (adjusted hazard ratio, 1.39 per 1-mm increase; 95% confidence interval, 1.09-1.78; p = 0.008). CONCLUSIONS: : Extracorporeal cardiopulmonary resuscitation can improve neurologic outcome after out-of-hospital cardiac arrest of cardiac origin; furthermore, pupil diameter on hospital arrival may be a key predictor to identify extracorporeal cardiopulmonary resuscitation candidates.

8. Outcome prediction by motor and pupillary responses in children treated with therapeutic hypothermia after cardiac arrest.

**Authors**	Abend NS; Topjian AA; Kessler SK; Gutierrez-Colina AM; Berg RA; Nadkarni V; Dlugos DJ; Clancy RR; Ichord RN; Abend, Nicholas S; Topjian, Alexis A; Kessler, Sudha Kilaru; Gutierrez-Colina, Ana M; Berg, Robert A; Nadkarni, Vinay; Dlugos, Dennis J; Clancy, Robert R; Ichord, Rebecca N
**Source**	Pediatric Critical Care Medicine; Jan 2012; vol. 13 (no. 1); p. 32-38
**Publication Date**	Jan 2012
**Publication Type(s)**	Academic Journal
**PubMedID**	21499174
**Database**	CINAHL<br>Available at Pediatric Critical Care Medicine from Ovid (LWW Total Access Collection 2015 - Q1 with Neurology)<br>Available at Pediatric Critical Care Medicine from PubMed Central

Abstract Objective: Clinical neurologic signs considered predictive of adverse outcome after pediatric cardiac arrest may have a different prognostic value in the setting of therapeutic hypothermia. We aimed to determine the prognostic value of motor and pupillary responses in children treated with therapeutic hypothermia after cardiac arrest.Design: Prospective cohort study.Setting: Pediatric intensive care unit in tertiary care hospital.Patients: Children treated with therapeutic hypothermia after cardiac arrest.Measurements and Main Results: Thirty-five children treated with therapeutic hypothermia after cardiac arrest were prospectively enrolled. Examinations were performed by emergency medicine physicians and intensive care unit bedside nurses. Examinations were performed after resuscitation, 1 hr after achievement of hypothermia, during the last hour of hypothermia, 1 hr after achievement of normothermia, after 24 hrs of normothermia, and after 72 hrs of normothermia. The primary outcome was unfavorable outcome at intensive care unit discharge, defined as a pediatric cerebral performance category score of 4-6 at hospital discharge. The secondary outcome was death (pediatric cerebral performance category = 6). The associations between exam responses and unfavorable outcomes (as both pediatric cerebral performance category 4, 5, 6 and pediatric cerebral performance category 6) are presented as positive predictive values, for both all subjects and subjects not receiving paralytics. Statistical significance for these comparisons was determined using Fisher's exact test. At all examination times and examination categories, positive predictive values were higher for the unfavorable outcome pediatric cerebral performance category 4, 5, 6 than the pediatric cerebral performance category 6. By normothermia hour 24, absent motor and pupil responses were highly predictive of unfavorable outcome (pediatric cerebral performance category 4, 5, 6) (positive predictive value 100% and $p < .03$ for all categories), while at earlier times the predictive value was lower.Conclusions: Absent motor and pupil responses are more predictive of unfavorable outcome when defined more broadly than when defined as only death. Absent motor and pupil responses during hypothermia and soon after return of spontaneous circulation were not predictive of unfavorable outcome while absent motor and pupil responses once normothermic were predictive of unfavorable short-term outcome. Further study is needed using more robust short-term and long-term outcome measures.

9. Looking into the crystal ball: Can we predict prognosis in children treated with therapeutic hypothermia after cardiac arrest?*.

Authors Pessach IM; Paret G
Source Pediatric Critical Care Medicine; Jan 2012; vol. 13 (no. 1); p. 97-98
Publication Date Jan 2012
Publication Type(s) Academic Journal
PubMedID 22222645
Database CINAHL
Available at Pediatric critical care medicine : a journal of the Society of Critical Care Medicine and the World Federation of Pediatric Intensive and Critical Care Societies from Ovid (LWW Total Access Collection 2015 - Q1 with Neurology)

10. Reversible brain death after cardiopulmonary arrest and induced hypothermia.

Authors Webb AC; Samuels OB
Source Critical Care Medicine; Jun 2011; vol. 39 (no. 6); p. 1538-1542
Publication Date Jun 2011
Publication Type(s) Academic Journal
PubMedID 21494112
Database CINAHL
Available at Critical care medicine from Ovid (LWW Total Access Collection 2015 - Q1 with Neurology)
Available at Critical care medicine from Ovid (Journals @ Ovid) - London Health Libraries

Abstract OBJECTIVE: : To describe a patient with transient reversal of findings of brain death after cardiopulmonary arrest and attempted therapeutic hypothermia. DESIGN: : Case report. SETTING: : Intensive care unit of an academic tertiary care hospital. PATIENT: : A 55-yr-old man presented with cardiac arrest preceded by respiratory arrest. Cardiopulmonary resuscitation was performed, spontaneous perfusion restored, and therapeutic hypothermia was attempted for neural protection. After rewarming to 36.5°C, neurologic examination showed no eye opening or response to pain, spontaneous myoclonic movements, sluggishly reactive pupils, absent corneal reflexes, and intact gag and spontaneous respirations. Over 24 hrs, remaining cranial nerve function was lost. The neurologic examination was consistent with brain death. Apnea test and repeat clinical examination after a duration of 6 hrs confirmed brain death. Death was pronounced and the family consented to organ donation. Twenty-four hrs after brain death pronouncement, on arrival to the operating room for organ procurement, the patient was found to have regained corneal reflexes, cough reflex, and spontaneous respirations. The care team faced the challenge of offering an adequate explanation to the patient's family and other healthcare professionals involved. INTERVENTIONS: : Induced hypothermia and brain death determination. MEASUREMENTS AND MAIN RESULTS: : This represents the first published report in an adult patient of reversal of a diagnosis of brain death made in full adherence to American Academy of Neurology guidelines. Although the reversal was transient and did not impact the patient's prognosis, it impacted his eligibility for organ donation and cast doubt about the ability to determine irreversibility of brain death findings in patients treated with hypothermia after cardiac arrest. CONCLUSIONS: : We strongly recommend caution in the determination of brain death after cardiac arrest when induced hypothermia is used. Confirmatory testing should be considered and a minimum observation period after rewarming before brain death testing ensues should be established.

11. Multicenter cohort study of out-of-hospital pediatric cardiac arrest.

Authors Moler FW; Donaldson AE; Meert K; Brilli RJ; Nadkarni V; Shaffner DH; Schleien CL; Clark RS; Dalton HJ; Statler K; Tieves KS; Hackbarth R; Pretzlaff R; van der Jagt EW; Pineda J; Hernan L; Dean JM; Moler, Frank W; Donaldson, Amy E; Meert, Kathleen

Source Critical Care Medicine; Jan 2011; vol. 39 (no. 1); p. 141-149

Publication Date Jan 2011

Publication Type(s) Academic Journal

PubMedID 20935561

Database CINAHL

Available at Critical Care Medicine from Ovid (LWW Total Access Collection 2015 - Q1 with Neurology)

Available at Critical Care Medicine from Ovid (Journals @ Ovid) - London Health Libraries

Available at Critical Care Medicine from PubMed Central

Abstract Objectives: To describe a large cohort of children with out-of-hospital cardiac arrest with return of circulation and to identify factors in the early postarrest period associated with survival. These objectives were for planning an interventional trial of therapeutic hypothermia after pediatric cardiac arrest.Methods: A retrospective cohort study was conducted at 15 Pediatric Emergency Care Applied Research Network clinical sites over an 18-month study period. All children from 1 day (24 hrs) to 18 yrs of age with out-of-hospital cardiac arrest and a history of at least 1 min of chest compressions with return of circulation for at least 20 mins were eligible.Measurements and Main Results: One hundred thirty-eight cases met study entry criteria; the overall mortality was 62% (85 of 138 cases). The event characteristics associated with increased survival were as follows: weekend arrests, cardiopulmonary resuscitation not ongoing at hospital arrival, arrest rhythm not asystole, no atropine or $NaHCO_3$, fewer epinephrine doses, shorter duration of cardiopulmonary resuscitation, and drowning or asphyxial arrest event. For the 0- to 12-hr postarrest return-of-circulation period, absence of any vasopressor or inotropic agent (dopamine, epinephrine) use, higher lowest temperature recorded, greater lowest pH, lower lactate, lower maximum glucose, and normal pupillary responses were all associated with survival. A multivariate logistic model of variables available at the time of arrest, which controlled for gender, age, race, and asystole or ventricular fibrillation/ventricular tachycardia anytime during the arrest, found the administration of atropine and epinephrine to be associated with mortality. A second model using additional information available up to 12 hrs after return of circulation found 1) preexisting lung or airway disease; 2) an etiology of arrest drowning or asphyxia; 3) higher pH, and 4) bilateral reactive pupils to be associated with lower mortality. Receiving more than three doses of epinephrine was associated with poor outcome in 96% (44 of 46) of cases.Conclusions: Multiple factors were identified as associated with survival after out-of-hospital pediatric cardiac arrest with the return of circulation. Additional information available within a few hours after the return of circulation may diminish outcome associations of factors available at earlier times in regression models. These factors should be considered in the design of future interventional trials aimed to improve outcome after pediatric cardiac arrest.

12. Predictors of neurologic outcome in hypothermia after cardiac arrest.

Authors Fugate JE; Wijdicks EF; Mandrekar J; Claassen DO; Manno EM; White RD; Bell MR; Rabinstein AA

Source Annals of Neurology; Dec 2010; vol. 68 (no. 6); p. 907-914

Publication Date Dec 2010

**Publication Type(s)**	Academic Journal
**PubMedID**	21061401
**Database**	CINAHL
	Available at Annals of neurology from Wiley Online Library Science , Technology and Medicine Collection 2017

Abstract OBJECTIVE: To evaluate the predictive value of neurologic prognostic indicators for patients treated with hypothermia after surviving cardiopulmonary arrest. METHODS: Patients who survived cardiopulmonary arrest were prospectively collected from June 2006 to October 2009. Detailed neurologic examinations were performed. Serum neuron specific enolase (NSE) measurements, brain imaging findings, somatosensory evoked potentials, and electroencephalogram (EEG) results were recorded. EEG patterns were blindly dichotomized with malignant patterns consisting of burst-suppression, generalized suppression, status epilepticus, and nonreactivity. Outcome measure of in-hospital mortality was assessed. RESULTS: A total of 192 patients (103 hypothermic, 89 nonhypothermic) were studied. The absence of pupillary light responses, corneal reflexes, and an extensor or absent motor response at Day 3 after cardiac arrest remained accurate predictors of poor outcome after therapeutic hypothermia ($p < 0.0001$ for all). Myoclonic status epilepticus was invariably associated with death ($p = 0.0002$). Malignant EEG patterns and global cerebral edema on head computed tomography (CT) were associated with death in both populations ($p < 0.001$). NSE > 33 ng/ml levels measured 1-3 days after cardiac arrest remained associated with poor outcome ($p = 0.017$), but had a false-positive rate of 29.3% (95% confidence interval [CI] 0.164-0.361). INTERPRETATION: Clinical examination (brainstem reflexes, motor response, and presence of myoclonus) at Day 3 after cardiac arrest remains an accurate predictor of outcome after therapeutic hypothermia. Sedative medications in both hypothermic and nonhypothermic patients may confound the clinical exam. NSE > 33 ng/ml has a high false-positive rate in patients treated with hypothermia and should be interpreted with caution.

13. Association between clinical examination and outcome after cardiac arrest.

**Authors**	Rittenberger JC; Sangl J; Wheeler M; Guyette FX; Callaway CW; Rittenberger, Jon C; Sangl, John; Wheeler, Matthew; Guyette, Francis X; Callaway, Clifton W
**Source**	Resuscitation; Sep 2010; vol. 81 (no. 9); p. 1128-1132
**Publication Date**	Sep 2010
**Publication Type(s)**	Academic Journal
**PubMedID**	20732605
**Database**	CINAHL
	Available at Resuscitation from PubMed Central

Abstract Background: Neurologic prognostication after cardiac arrest relies on clinical examination findings derived before the advent of therapeutic hypothermia (TH). We measured the association between clinical examination findings at hospital arrival, 24, and 72 h after cardiac arrest in a modern intensive care unit setting.Methods: Between 1/1/2005 and 3/31/2009, hospital charts were reviewed in 272 subjects for neurologic examination findings (Glasgow Coma Score--motor examination, pupil response, corneal response) at hospital arrival, 24, and 72 h following cardiac arrest. Primary outcome was survival to hospital discharge. Secondary outcome was "good outcome," defined as discharge to home or acute rehabilitation facility.Results: Mean age was 61 years; 155 (57%) were male. Most were treated with TH (N=161; 59%) and 100 subjects (37%) were in ventricular fibrillation/ventricular tachycardia. Out-of-hospital cardiac arrest was common (N=169; 62%). Ninety-one (33%) survived, with 54 (20%) experiencing a good outcome. In subjects with a GCS Motor score < or = 3 at 24 and 72 h survival was 17% (13/76; 95% CI 7.9-26.2%) and 20% (6/27; 95% CI 6.3-33.6%), respectively. Subjects with a GCS Motor score < or = 2 at 24 and 72 h survived in 14% (9/66; 95% CI 4.6-22.6%) and 18% (6/33; 95% CI 3.5-32.8%), respectively. Absent pupil reactivity on arrival did not exclude survival (7/65; 11%; 95% CI 2.4-19%). A lack of pupil reactivity or corneal response at 72 h was associated with death (pupil: 0/17; 95% CI 0, 2.9%; corneal: 0/21; 95% CI 0, 2.4%).Conclusions: GCS Motor score < or = 3 or < or = 2 at 24 or 72 h following cardiac arrest does not exclude survival or good outcome. However, absent pupil or corneal response at 72 h appears to exclude survival and good outcome.

14. Predictors of poor neurologic outcome after induced mild hypothermia following cardiac arrest...Neurology. 2008 Nov 4;71(19):1535-7

**Authors**	Freeman WD; Barrett KM; Biewend ML; Johnson MM; Divertie GD; Meschia JF; Bryan Young Faan G
**Source**	Neurology; Sep 2009; vol. 73 (no. 12); p. 997-998
**Publication Date**	Sep 2009
**Publication Type(s)**	Academic Journal
**PubMedID**	19770479
**Database**	CINAHL

15. A clinical and EEG scoring system that predicts early cortical response (N20) to somatosensory evoked potentials and outcome after cardiac arrest.

**Authors**	Daubin C; Guillotin D; Etard O; Gaillard C; du Cheyron D; Ramakers M; Bouchet B; Parienti JJ; Charbonneau P
**Source**	BMC Cardiovascular Disorders; Jan 2008; vol. 8 ; p. 35-35

Publication Date Jan 2008
Publication Type(s) Academic Journal
PubMedID 19055810
Database CINAHL
Available at BMC Cardiovascular Disorders from BioMed Central
Available at BMC Cardiovascular Disorders from Europe PubMed Central - Open Access
Available at BMC Cardiovascular Disorders from ProQuest (Hospital Premium Collection) - NHS Version
Available at BMC Cardiovascular Disorders from biomedcentral.com

Abstract Anoxic coma following cardiac arrest is a common problem with ethical, social, and legal consequences. Except for unfavorable somatosensory-evoked potentials (SSEP) results, predictors of unfavorable outcome with a 100% specificity and a high sensitivity are lacking. The aim of the current research was to construct a clinical and EEG scoring system that predicts early cortical response (N20) to somatosensory evoked potentials and 6-months outcome in comatose patients after cardiac arrest.~Background~Background~We retrospectively reviewed the records of all consecutive patients who suffered cardiac arrest outside our hospital and were subsequently admitted to our facility from November 2002 to July 2006. We scored each case based on early clinical and EEG factors associated with unfavorable SSEPs, and we assessed the ability of this score to predict SSEP results and outcome.~Methods~Methods~Sixty-six patients qualified for inclusion in the cohort. Among them, 34 (52%) had unfavorable SSEP results. At day three, factors independently associated with unfavorable SSEPs were: absence of corneal (14 points) and pupillary (21 points) reflexes, myoclonus (25 points), extensor or absent motor response to painful stimulation (28 points), and malignant EEG (11 points). A score >40 points had a sensitivity of 85%, a specificity of 84%, and a positive predictive value (PPV) of 85% to predict unfavorable SSEP results. A score >88 points had a PPV of 100%, but a sensitivity of 18%. Overall, this score had an area under ROC curves of 0.919. In addition, at day three, a score > 69 points had a PPV of 100% with a sensitivity of 32% to predict death or vegetative state.~Results~Results~A scoring system based on a combination of clinical and EEG findings can predict the absence of early cortical response to SSEPs. In settings without access to SSEPs, this score may help decision-making in a subset of comatose survivors after a cardiac arrest.~Conclusion~Conclusions

16. Survival from cardiac arrest in the Accident and Emergency Department.

Authors Cope, A R; Quinton, D N; Dove, A F; Sloan, J P; Dave, S H
Source Journal of the Royal Society of Medicine; Dec 1987; vol. 80 (no. 12); p. 746-749
Publication Date Dec 1987
Publication Type(s) Academic Journal
PubMedID 3430528
Database CINAHL
Available at Journal of the Royal Society of Medicine from Europe PubMed Central - Open Access
Available at Journal of the Royal Society of Medicine from PubMed Central

Abstract One hundred consecutive patients who were treated in an Accident and Emergency Department for 'cardiac arrest' were studied prospectively. Of these 30% had arrested within the community, 21% in transit and 49% in hospital. The immediate outcome was that 40 left the A&E Department alive; of these, 13 left hospital alive. The 'survivors' included 3 cases of documented asystole. Patients who were over the age of 65, who arrested out of hospital and at night, were found to have a poor prognosis. The time between arrest and arrival of the ambulance was found to affect outcome. Patients with ventricular fibrillation had the best prognosis and those with electromechanical dissociation the worst.

17. Clinical reasoning: Prognostication after cardiac arrest: What do we really know?

Authors Beekman R.B.; Greer D.M.; Maciel C.B.; Brooks D.C.
Source Neurology; 2017; vol. 89 (no. 20)
Publication Date 2017
Publication Type(s) Note
Database EMBASE

18. Prognostic impact of coagulopathy at hospital admission on 30-day neurological outcomes in patients with out-of-hospital cardiac arrest

Authors Murakami N.; Kokubu N.; Kamada Y.; Noto T.; Nagano N.; Nishida J.; Miura T.; Tsuchihashi K.; Narimatsu E.
Source European Heart Journal; Aug 2017; vol. 38 ; p. 591
Publication Date Aug 2017
Publication Type(s) Conference Abstract
Database EMBASE
Available at European Heart Journal from Oxford Journals - Medicine

Abstract Background: It has been reported that systemic coagulopathy develops in patients resuscitated after out-of hospital cardiac arrest (OHCA). Although one of the major causes of high mortality rate in the OHCA patients is disseminated intravascular coagulation, the prognostic implication of coagulopathy at hospital admission has not been clarified. Here we investigated this issue by data from consecutive OHCA patients. Methods: From 2010 to 2015, consecutive 1630 patients with endogenous origin OHCA admitted to the emergency room in our institute. Of these patients, 174 patients were treated in the coronary care unit, of whom 85 patients received extracorporeal cardiopulmonary resuscitation (ECPR). We analyzed data regarding coagulation and fibrinolytic factors at hospital admission: international normalized ratio of prothrombin time (PT-INR), activated partial thromboplastin time (APTT), antithrombin (AT), plasma fibrinogen (FBG) and fibrinogen degradation products (FDP). ECPR was performed in OHCA patients with refractory ventricular fibrillation that was not terminated by conventional CPR. Targeted temperature management was performed in unconscious and stable hemodynamic patients after return of spontaneous circulation or ECPR implement; target core temperature was 34degreeC and was maintained for 24 hours. Thirty-day neurological outcome was evaluated using cerebral performance category (CPC), and favorable recovery and unfavorable recovery were defined as CPC 1-2 and CPC 3-5, respectively. Results: Of 174 OHCA patients, 108 patients (62%) were survived, and 69 patients (40%) showed favorable recovery. PT-INR (1.3+/-0.6 vs. 1.6+/-0.9, p<0.05), FDP (13+/-20 vs. 23+/-36 mug/ml, p<0.05) and APTT (35+/-19 vs. 60+/-40 sec, p<0.05) were significantly lower in the favorable recovery group than those in the unfavorable recovery group. Furthermore, AT (88+/-15 vs. 77+/-19%, p<0.05) in favorable recovery group was significantly higher compared to that in the unfavorable recovery group. There was no significant difference in FBG between these two groups. Absence of asystole at the first monitoring, high Glasgow Coma Scale (GCS), pupillary reflex and low APTT (OR 0.98, 95% CI 0.96-0.99, p<0.05) were shown to be significantly associated with favorable neurological recovery by the results of multivariate logistic regression analysis using first monitored asystole, OHCA caused by acute myocardial infarction, GCS, pupillary reflex, APTT and FDP as explanatory variables. The explanatory variables for favorable recovery were selected by the stepwise method based on Akaike's information criterion. In receiver operating characteristic curve analysis, optimal cut-off value of APTT to predict favorable neurological outcome was 35.1 sec, of which sensitivity and specificity were 75% and 56%, respectively (area under curve 0.76, p<0.05). Conclusion: In patients with OHCA, APTT at hospital admission may be a one of independent predictors of neurological recovery in OHCA patients.

19. Multimodal Outcome Prognostication After Cardiac Arrest and Targeted Temperature Management: Analysis at 36 degreeC

Authors Tsetsou S.; Novy J.; Rossetti A.O.; Pfeiffer C.; Oddo M.
Source Neurocritical Care; Feb 2018; vol. 28 (no. 1); p. 104-109
Publication Date Feb 2018
Publication Type(s) Article
Database EMBASE
Available at Neurocritical care from SpringerLink

Abstract Background: Targeted temperature management (TTM) represents the standard of care in comatose survivors after cardiac arrest (CA) and may be applied targeting 33degree or 36 degreeC. While multimodal prognostication has been extensively tested for 33 degreeC, scarce information exists for 36 degreeC. Methods: In this cohort study, consecutive comatose adults after CA treated with TTM at 36 degreeC between July 2014 and October 2016 were included. A combination of neurological examination, electrophysiological features, and serum neuron-specific enolase (NSE) was evaluated for outcome prediction at 3 months (mortality; good outcome defined as cerebral performance categories (CPC) score of 1-2, poor outcome defined as CPC 3-5). Results: We analyzed 61 patients. The presence of two or more predictors out of, unreactive electroencephalogram (EEG) background, epileptiform EEG, absent pupillary and/or corneal reflex, early myoclonus, bilaterally absent cortical somatosensory evoked potentials, and serum NSE >75 mug/l, had a high specificity for predicting mortality (positive predictive value [PPV] = 1.00, 95% CI 0.87-1.00) and poor outcome (PPV = 1.00, 95% CI 0.80-1.00). Reactive EEG background was highly sensitive for predicting good outcome (0.95, 95% CI 0.74-0.99). Conclusions: Prediction of outcome after CA and TTM targeting 36 degreeC seems valid in adults using the same features tested at 33 degreeC. A reactive EEG under TTM appears highly sensitive for good outcome.
Copyright © 2017, Springer Science+Business Media New York.

20. Predictors of functional outcome after hanging injury

Authors Kao C.-L.; Hsu I.-L.
Source Chinese Journal of Traumatology - English Edition; 2018
Publication Date 2018
Publication Type(s) Article In Press
Database EMBASE
Available at Chinese Journal of Traumatology from Europe PubMed Central - Open Access

Abstract Purpose: Suicide becomes a serious problem in today's society and hanging is a common method of suicide. We want to find the factors which can predict the final functional outcomes of these cases. Methods: All patients who presented to Accident and Emergency Department (ED) of the National Cheng Kung University Hospital from 1st January 2005 to 31th December 2013 with a hanging injury were included in this study. All cases were divided into good outcome group and bad outcome group according to Glasgow Outcome Scale (GOS). Data was analyzed by Mann-Whitney test and chi-square test. Results: Glasgow Coma Scale (GCS) < 3, pupil dilation and no pupillary light reflex both at the scene and ED were the factors to indicate poor functional outcome. Out-hospital cardiac arrest (OHCA), acidosis (pH < 7.2) and the need for intubation once arriving at ED were also related to poor functional outcome. OHCA cases all had poor functional outcome. Conclusion: GCS, pupil size, pupillary light reflex, OHCA and acidosis are useful as prognostic factors. GCS = 3 lead to a very poor outcome. However, the functional outcome seems good in patients with GCS>3. There parameters can help to predict the outcome before treatment.
Copyright © 2018.

21. Role of electrophysiologic studies in the first 72 hours in comatose postcardiac arrest patients

Authors Okada M.; Kashima Y.; Takeshige K.; Imamura H.
Source Critical Care Medicine; Jan 2018; vol. 46 ; p. 139
Publication Date Jan 2018
Publication Type(s) Conference Abstract
Database EMBASE
Available at Critical Care Medicine from Ovid (Journals @ Ovid) - London Health Libraries
Abstract Learning Objectives: Electrophysiological studies have been proposed for use in the predictors of poor neurological outcome in adult comatose survivors of cardiac arrest. However, role of these studies in patients with comatose out-of-hospital cardiac arrest (OHCA) within the first 72 h during TTM remains unclear. Therefore, we compared the prognostic significance of somatosensory evoked potentials (SSEP), auditory brainstem response (ABR), and electroencephalogram (EEG) for neurological outcome in comatose OHCA patients treated with targeted temperature management (TTM). Methods: We enrolled 76 comatose OHCA patients and performed SSEP, ABR and EEG within 72 hours after onset of cardiac arrest during TTM. EEGs were classified into highly malignant (suppression, suppression with periodic discharges, burst-suppression), malignant (periodic or rhythmic patterns, pathological or nonreactive background), and benign EEG (absence of malignant features). Good or poor neurological outcome was defined as Cerebral Performance Categories 1-2 vs. 3-5 at discharge. Results: All patients whose N20 wave of SSEP (n = 40) were poor neurological outcome (SSEP; sensitivity, 0.784; specificity, 1.0; positive predictive value (PPV), 1.0; negative predictive value (NPV), 0.694). All patients whose V wave of ABR (n = 11) were dead (ARB; sensitivity, 0.216; specificity, 1.0; PPV, 1.0; NPV, 0.385); however, the absence of V wave of ARB was associated with loss of brain stem reaction, including changes in a pupil diameter. Prognostic values of EEG for poor neurological outcome were: highly malignant EEG group (n = 42; sensitivity, 0.824; specificity, 0.96; PPV, 0.977; NPV, 0.727), highly malignant or malignant EEG group (n = 51; sensitivity, 0.902; specificity, 0.96; PPV, 0.979; NPV, 0.828), respectively. Conclusions: Although several reports indicate that SSEP does not exit 100% reliability in diagnostic, physical examination could substitute for ARB, and EEG, even if highly malignant pattern, was not able to predict poor outcome completely, electrophysiological studies reliably predicted poor neurological outcome during TTM. Additionally, EEG could detect status epilepticus masked by use of sedative drugs or muscle-relaxant anesthetics during TTM. The role of electrophysiological studies in predicting neurological outcome after OHCA might be limited within first 72 h after OHCA; however, electrophysiological studies were useful for not only predicted outcome but also neurocritical care in the era of TTM.

22. Very early pupillometry, eeg suppression, and bis data predict outcome 6 hours after cardiac arrest

Authors Sawyer M.; Riker R.; May T.; Fischman V.; Stone P.; Eldridge A.; Lord C.; Lucas F.; Seder D.
Source Critical Care Medicine; Jan 2018; vol. 46 ; p. 126
Publication Date Jan 2018
Publication Type(s) Conference Abstract
Database EMBASE
Available at Critical Care Medicine from Ovid (Journals @ Ovid) - London Health Libraries

Abstract Learning Objectives: Predicting outcome after cardiac arrest is complicated; current guidelines recommend delaying prognostication at least 72 hours after return of spontaneous circulation (ROSC). Earlier methods to accurately quantify severity of injury are needed to stratify hypoxic-ischemic encephalopathy, facilitating enrollment in cardiac arrest treatment trials. Quantitative EEG and pupillometry techniques may provide very early prognosis information. Methods: Data from adult patients treated with Targeted Temperature Management (TTM) after cardiac arrest were prospectively entered into the International Cardiac Arrest Registry (INTCAR) database. Data from the Neuroptics NPi-200 pupillometer (Neurological Pupil index-NPi, constriction velocity-CV, percent constriction-%C) and the bispectral index (BIS), and suppression ratio (SR) were recorded at start of TTM and 6 hours after ROSC. Continuous EEG was interpreted by Neurologists and categorized as malignant EEG Pattern, Burst Suppression, or Other. Discharge outcomes were defined as Good if Cerebral Performance Category Score was 1 or 2, and Poor (PO) if 3-5. Receiver Operator Characteristic curves were constructed to predict PO. Results: 55 patients were enrolled, with a median age of 57 years; 35 patients were male (65%). 37 patients (69%) had a PO predicted by peak Neuron Specific Enolase (NSE) levels (AUC=0.90, $p < 0.001$) and malignant EEG features (0.81, $p < 0.001$) within 72 hours of ROSC. Very early predictors of PO included initial BIS (0.89, $p < 0.001$), SR (0.85, $p < 0.001$), and NPi (0.70, $p = 0.003$) and 6 hour CV (0.73, $p = 0.002$) and %C (0.68, $p = 0.01$). Spearman correlation was strong between NSE and initial BIS ($r=0.77$, $p < 0.001$) and SR (0.75, $p < 0.001$), less so for NPi (0.37, $p = 0.01$). Combining initial BIS and NPi also predicted PO (AUC=0.90, $p < 0.001$). Conclusions: Although EEG features and NSE values in the first 72 hours after ROSC can predict outcome after cardiac arrest as recommended by the American Heart Association in 2015, very early monitoring with bispectral index, suppression ratio, and quantitative pupillometry also appear accurate predictors. These tools may provide accurate very early stratification of brain injury, facilitating appropriate enrollment in therapeutic trials after cardiac arrest.

23. Cerebral vasomotor reactivity test during targeted temperature management is feasible for prognostication after out of hospital cardiac arrest

Authors Lee S.E.; Chae M.K.; Hong J.M.
Source Circulation; Nov 2017; vol. 136
Publication Date Nov 2017
Publication Type(s) Conference Abstract
Database EMBASE

Abstract Background: Neurological prognostication after cardiac arrest is a complex process requiring a multimodal approach. Recently, some reports presented that cerebral autoregulation (CA) was labile to hypoxic brain damage and impaired CA was correlated with neurologically poor outcome. The aim of this study was to determine whether vasomotor reactivity (VMR) test by transcranial doppler (TCD), reflecting cerebral hemodynamic status, was feasible for neurological prognostication in post cardiac arrest patients. Methods: From October 2015 to February 2017, a total of 21 patients with VMR tests as well as conventional tests, such as EEG and SEP were enrolled. Primary outcome was cerebral performance category scale (CPC) at discharge. The VMR test was done by a breath-holding method during 40 seconds with neuromuscular blockade and a pause in ventilation and recorded changes of mean flow velocity on monitored vessel by TCD measurements. The VMR test was done on the first day in 24 hours of ROSC. Other conventional prognostication tests such as electroencephalography (EEG), somatosensory evoked potential (SEP), et al., was performed after 72 hours from ROSC. We divided patients between good (CPC 1-2, n=7) and poor (CPC 3-5, n=14) outcome group and compared results from prognostic tests between two groups. Results: As previous studies, impaired pupillary light reflex, absent N20 at median nerve SEP, and malignant pattern on EEG after 72 hours from ROSC were presented as reliable results in poor outcome group. ($p=0.047$, $p<0.001$, $p<0.001$, respectively) Significant impaired VMR (increment of mean flow velocity < 20%) during TTM period also was more frequent in the poor outcome group. (85.7% vs. 0.0%, $p<0.001$) In receiver operating characteristic (ROC) analysis for prognostication of poor outcome, all neurophysiologic test has high area under the curve (AUC). (EEG vs. SEP vs. VMR; 0.929 vs. 0.964 vs. 0.929) If abnormal findings was noted on more than two study among EEG, SEP, and VMR, AUC was noted on 1.000. Conclusions: The present study shows that vasomotor reactivity is impaired in patients with neurological poor outcome after cardiac arrest. Cerebral hemodynamic status evaluation by VMR seems to be feasible tool for early prognostication after cardiac arrest.

24. Automated pupillometry for early prognostication in comatose cardiac arrest patients: Preliminary results of a multicenter study

Authors Oddo M.; Cariou A.; Citerio G.; Friberg H.; Horn J.; Payen J.-F.; Sandroni C.; Stammet P.; Storm C.; Taccone F.S.
Source Intensive Care Medicine Experimental; Sep 2017; vol. 5 (no. 2)
Publication Date Sep 2017
Publication Type(s) Conference Abstract
Database EMBASE
Available at Intensive care medicine experimental from Europe PubMed Central - Open Access
Available at Intensive care medicine experimental from International DOI Foundation

Abstract INTRODUCTION. Bilateral absence of pupillary reactivity 3 days after cardiac arrest (CA) predicts poor outcome, however prognostic accuracy of standard examination may be lower when performed at an earlier phase and is limited by lack of a quantitative tool. OBJECTIVES. To examine the accuracy of quantitative pupillometry (NPi-200 automated infrared pupillometer, NeurOptics, Irvine, CA, USA) in predicting neurological recovery of post-CA coma and to compare its prognostic value to that of standard qualitative neurological examination. METHODS. A prospective multicentre study was conducted amongst 10 European academic hospitals (clinicaltrials.gov NCT02607878). Blinded pupillometry tests (Neurological Pupil index [NPi] and % of Pupillary Light Reactivity [PLR]) were performed in parallel with standard neurological examination (motor response [GCS-M] and brainstem reflexes [BSR]) at day 1 and 2 after CA. Outcome was assessed at 3 months using the Cerebral Performance Categories (CPC) score; poor outcome was defined as CPC 4 (vegetative state) and 5 (death). Statistical analyses were performed by an independent statistician. RESULTS. From March 2015 to October 2016, 371 consecutive patients were included in this ongoing study. On day 1, patients with poor outcome (n = 206; 56%) had lower NPi (3.6 +/- 1.5 vs. 4.5 +/- 0.3, p < 0.001) and lower PLR (13 [8, 19] vs. 20% [17, 26], p < 0.001) than patients with good outcome (n = 165; 44%). Similar results were found on day 2. Low NPi (<3) and PLR (<13%) had higher specificity and positive predictive value for predicting 3-month poor outcome than absent BSR and GCS-M, on both day 1 and day 2 after CA (Table 6). CONCLUSIONS. These findings indicate that quantitative pupillometry had higher accuracy than standard qualitative neurological examination in predicting poor outcome in the early ICU phase after CA. Automated infrared pupillometry may be integrated in multimodal algorithms for coma prognostication following CA. (Table presented).

25. Neurological pupil index predicts neurological outcome early after cardiac arrest: An observational study

Authors Sawyer M.E.; Lucas L.; May T.; Lord C.; Seder D.B.; Riker R.R.
Source Neurocritical Care; 2017; vol. 27 (no. 2)
Publication Date 2017
Publication Type(s) Conference Abstract
Database EMBASE
Available at Neurocritical care from SpringerLink

Abstract Introduction The pupillary light reflex is associated with outcome after cardiac arrest as a dichotomous variable (present/absent) at various time points following resuscitation (ROSC). Infrared pupillometry provides quantitative measures including pupil diameter (PD), and Neurological Pupil Index (NPi) which ranges from 0 (nonreactive) to 5 (brisk) and reflects velocity and degree of pupil constriction in response to a standardized light stimulus. These measures may provide early prognostic information to guide therapy. Methods Comatose adult survivors of cardiac arrest treated with targeted temperature management were monitored with the Neuroptics NPi-200 pupillometer. Outcomes were defined as good (GO) if discharge Cerebral Performance Category score was 1-2, and poor (PO) if 3-5. Data are presented as median (IQR). Groups were compared using non-parametric statistical tests. Results Fifty-one patients were enrolled; the median age was 57 (48.5-68.5), and 33 (65%) were male. Initial rhythm was VT/VF in 55%, asystole in 23%, and PEA in 20%. Outcome was good in 16 (31%) patients. The initial PD did not differ between outcome groups [3.1 (2-4.7) PO vs 3.0 (2-4.2) GO]. The initial NPi was lower in poor outcome patients [3.3 (1.5-4) vs 3.9 (2.4-4.2) GO, p=0.005] measured 4.5 (3.4-6.3) hours after ROSC. NPi dropped below 3 in more poor outcome patients [27(77%) vs 6(37.5%) GO, p=0.015], and to zero in 18(51%) poor vs 1(6%) good outcome patients (p=0.005). Receiver operator characteristic curves confirmed that initial NPi predicted poor outcome better than pupil diameter (AUC 0.78 vs 0.61, p=0.016). Conclusions A low Neurological Pupil index predicted poor outcome 4-6 hours after resuscitation from cardiac arrest, and dropped to abnormal levels (<3) and to zero (reflecting a non-reactive pupil) more often in patients with poor outcomes. Additional research is needed to define potential confounders, optimal timing, and thresholds for different levels of neurological risk with pupillometry.

26. Post-anoxic quantitative MRI changes may predict emergence from coma and functional outcomes at discharge

Authors Reynolds A.S.; Matthews E.; Roh D.J.; Park S.; Claassen J.; Elkind M.S.V.; Agarwal S.; Guo X.; Zhao B.; Brodie D.; Rabbani L.E.
Source Resuscitation; Aug 2017; vol. 117 ; p. 87-90
Publication Date Aug 2017
Publication Type(s) Article
Database EMBASE

Abstract Background Traditional predictors of neurological prognosis after cardiac arrest are unreliable after targeted temperature management. Absence of pupillary reflexes remains a reliable predictor of poor outcome. Diffusion-weighted imaging has emerged as a potential predictor of recovery, and here we compare imaging characteristics to pupillary exam. Methods We identified 69 patients who had MRIs within seven days of arrest and used a semi-automated algorithm to perform quantitative volumetric analysis of apparent diffusion coefficient (ADC) sequences at various thresholds. Area under receiver operating characteristic curves (ROC-AUC) were estimated to compare predictive values of quantitative MRI with pupillary exam at days 3, 5 and 7 post-arrest, for persistence of coma and functional outcomes at discharge. Cerebral Performance Category scores of 3-4 were considered poor outcome. Results Excluding patients where life support was withdrawn, >=2.8% diffusion restriction of the entire brain at an ADC of <=650 x 10^{-6} m^2/s was 100% specific and 68% sensitive for failure to wake up from coma before discharge. The ROC-AUC of ADC changes at <=450 x 10^{-6} mm^2/s and <=650 x 10^{-6} mm^2/s were significantly superior in predicting failure to wake up from coma compared to bilateral absence of pupillary reflexes. Among survivors, >0.01% of diffusion restriction of the entire brain at an ADC <=450 x 10^{-6} m^2/s was 100% specific and 46% sensitive for poor functional outcome at discharge. The ROC curve predicting poor functional outcome at ADC <=450 x 10^{-6} mm^2/s had an AUC of 0.737 (0.574-0.899, p = 0.04). Conclusion Post-anoxic diffusion changes using quantitative brain MRI may aid in predicting persistent coma and poor functional outcomes at hospital discharge.
Copyright © 2017 Elsevier B.V.

27. Pain-related Somato Sensory Evoked Potentials: A potential new tool to improve the prognostic prediction of coma after cardiac arrest

Authors Zanatta P.; Bosco E.; Sorbara C.; Linassi F.; Mazzarolo A.P.; Arico M.; Bendini M.; Ori C.; Carron M.; Scarpa B.
Source Critical Care; Nov 2015; vol. 19 (no. 1)
Publication Date Nov 2015
Publication Type(s) Article
PubMedID 26573633
Database EMBASE
Available at Critical Care from BioMed Central
Available at Critical Care from Europe PubMed Central - Open Access
Available at Critical Care from PubMed Central

Abstract Introduction: Early prediction of a good outcome in comatose patients after cardiac arrest still remains an unsolved problem. The main aim of the present study was to examine the accuracy of middle-latency SSEP triggered by a painful electrical stimulation on median nerves to predict a favorable outcome. Methods: No- and low-flow times, pupillary reflex, Glasgow motor score and biochemical data were evaluated at ICU admission. The following were considered within 72h of cardiac arrest: highest creatinine value, hyperthermia occurrence, EEG, SSEP at low- (10mA) and high-intensity (50mA) stimulation, and blood pressure reactivity to 50mA. Intensive care treatments were also considered. Data were compared to survival, consciousness recovery and 6-month CPC (Cerebral Performance Category). Results: Pupillary reflex and EEG were statistically significant in predicting survival; the absence of blood pressure reactivity seems to predict brain death within 7days of cardiac arrest. Middle- and short-latency SSEP were statistically significant in predicting consciousness recovery, and middle-latency SSEP was statistically significant in predicting 6-month CPC outcome. The prognostic capability of 50mA middle-latency-SSEP was demonstrated to occur earlier than that of EEG reactivity. Conclusions: Neurophysiological evaluation constitutes the key to early information about the neurological prognostication of postanoxic coma. In particular, the presence of 50mA middle-latency SSEP seems to be an early and reliable predictor of good neurological outcome, and its absence constitutes a marker of poor prognosis. Moreover, the absence 50mA blood pressure reactivity seems to identify patients evolving towards the brain death.
Copyright © 2015 Zanatta et al.

28. Early Absent Pupillary Light Reflexes after Cardiac Arrest in Patients Treated with Therapeutic Hypothermia

Authors Dhakal L.P.; Freeman M.L.; Ng L.K.; Freeman W.D.; Dimberg E.L.; Sen A.; Stanko C.M.; Rawal B.; Heckman M.G.; Hoyne J.B.; Rabinstein A.A.
Source Therapeutic Hypothermia and Temperature Management; Aug 2016; vol. 6 (no. 3); p. 116-121
Publication Date Aug 2016
Publication Type(s) Article
PubMedID 27135180
Database EMBASE

Abstract Loss of pupillary light reactivity is one recognized indicator of poor prognosis after cardiopulmonary resuscitation (CPR). However, drug overdose, low cardiac output, and/or resuscitation drugs can lead to impaired pupillary light reflex. To investigate pupillary light reflex status before therapeutic hypothermia (TH) in relation to neurological outcome, we retrospectively reviewed the data of a prospectively implemented TH protocol in patients with cardiac arrest (CA) at Mayo Clinic in Jacksonville, Florida (January 2006-January 2012), and Mayo Clinic in Scottsdale, Arizona (August 2010-March 2014). During this period, all CA patients who underwent hypothermia were included. These patients were selected from an institutional database and hypothermia data set. The Cerebral Performance Category (CPC) at time of discharge was our primary outcome measure. A CPC of 1 to 2 was defined as good outcome and a CPC from 3 to 5 was defined as poor outcome. We identified 99 patients who had CA treated with TH. Twenty-nine patients (29%) had pupils that were nonreactive to light on admission examination before TH, eight of whom later had return of pupil reactivity by day 3. Two of these 29 patients (6.9%) had good outcome, compared to 24 of 70 patients (34.3%) with pupils that were reactive to light (p = 0.005). Both of these patients had CA after illicit drug overdose. Early nonreactive pupils occurred in almost a third of patients after CPR and before TH in our patient population. Recovery of pupillary light reactivity is possible, and in a small minority of those cases (particularly when CA is preceded by the use of illicit drugs), a good outcome can be achieved.
Copyright © 2016 Mary Ann Liebert, Inc.

29. Neuron specific enolase and Glasgow motor score remain useful tools for assessing neurological prognosis after out-of-hospital cardiac arrest treated with therapeutic hypothermia

Authors Roger C.; Palmier L.; Louart B.; Claret P.-G.; de la Coussaye J.-E.; Lefrant J.-Y.; Muller L.; Molinari N.
Source Anaesthesia Critical Care and Pain Medicine; Aug 2015; vol. 34 (no. 4); p. 231-237
Publication Date Aug 2015
Publication Type(s) Article
PubMedID 26324761
Database EMBASE

Abstract Aim of the study Identifying clinical, electrophysiological and biological predictors for 6-month neurological outcome in survivors at day 3 after cardiac arrest (CA) treated with therapeutic hypothermia (TH). Methods We conducted a retrospective cohort study of adults comatose after out-of hospital CA treated with TH. All data were collected from medical charts and laboratory files. Results Between January 2010 and March 2013, among the 130 analysed CA survivors, 27 (21%) had a good neurological outcome at 6 months and 103 (79%) had a poor neurological outcome, including 98 deaths. The Glasgow coma score motor response (GCS-M), pupillary reflexes and Neuron Specific Enolase (NSE) were the three best predictors of neurological outcome (P < 0.0001). The area under the Receiver Operating Characteristic curve for NSE was 0.92 [0.84-0.99]. Conclusion NSE values, GCS-M scores and pupillary reflexes are the best predictors of poor 6-month outcome after out-of-hospital CA treated with TH. Of these, NSE values have the best-isolated prognostic performance when above 28.8 mug/L.
Copyright © 2015 Societe francaise d'anesthesie et de reanimation (Sfar)

30. Prospective Cohort Study Evaluating the Prognostic Value of Simple EEG Parameters in Postanoxic Coma

Authors Azabou E.; Vaugier I.; Annane D.; Sharshar T.; Lofaso F.; Fischer C.; Mauguiere F.
Source Clinical EEG and Neuroscience; Jan 2016; vol. 47 (no. 1); p. 75-82
Publication Date Jan 2016
Publication Type(s) Article
PubMedID 26545818
Database EMBASE
Available at Clinical EEG and Neuroscience from ProQuest (Hospital Premium Collection) - NHS Version

Abstract We prospectively studied early bedside standard EEG characteristics in 61 acute postanoxic coma patients. Five simple EEG features, namely, isoelectric, discontinuous, nonreactive to intense auditory and nociceptive stimuli, dominant delta frequency, and occurrence of paroxysms were classified yes or no. Sensitivity, specificity, positive predictive value (PPV), negative predictive value (NPV), and area under the receiver operating characteristic curve (AUC) of each of these variables for predicting an unfavorable outcome, defined as death, persistent vegetative state, minimally conscious state, or severe neurological disability, as assessed 1 year after coma onset were computed as well as Synek's score. The outcome was unfavorable in 56 (91.8%) patients. Sensitivity, specificity, PPV, NPV, and AUC of nonreactive EEG for predicting an unfavorable outcome were 84%, 80%, 98%, 31%, and 0.82, respectively; and were all very close to the ones of Synek score >3, which were 82%, 80%, 98%, 29%, and 0.81, respectively. Specificities for predicting an unfavorable outcome were 100% for isoelectric, discontinuous, or dominant delta activity EEG. These 3 last features were constantly associated to unfavorable outcome. Absent EEG reactivity strongly predicted an unfavorable outcome in postanoxic coma, and performed as accurate as a Synek score >3. Analyzing characteristics of some simple EEG features may easily help nonneurophysiologist physicians to investigate prognostic issue of postanoxic coma patient. In this study (a) discontinuous, isoelectric, or delta-dominant EEG were constantly associated with unfavorable outcome and (b) nonreactive EEG performed prognostic as accurate as a Synek score >3.
Copyright © EEG and Clinical Neuroscience Society (ECNS) 2015.

31. Clinical and EEG characteristics of patients with preserved cortical SSEPS after post-anoxic coma

Authors Nobile L.; Pognuz E.; Rossetti A.; Francesca V.; Gaspard N.; Vincent J.-L.; Oddo M.; Taccone F.
Source Critical Care Medicine; Dec 2016; vol. 44 (no. 12); p. 141
Publication Date Dec 2016
Publication Type(s) Conference Abstract
Database EMBASE
Available at Critical Care Medicine from Ovid (LWW Total Access Collection 2015 - Q1 with Neurology)
Available at Critical Care Medicine from Ovid (Journals @ Ovid) - London Health Libraries
Abstract Learning Objectives: Bilateral absence of a cortical response to somatosensory evoked potentials (SSEPs) is an established marker of poor neurological outcome (PNO) after cardiac arrest (CA). However, presence of these responses (N20PRES) does not reliably predict good neurological outcome (GNO). Few data are available on the clinical, electrophysiological and biochemical characteristics of N20PRES patients with PNO. Methods: In this retrospective analysis of institutional databases (2007-2014) from 3 academic ICUs, we included all comatose post-CA adult patients who had been treated with targeted temperature management for 24h, in whom multimodal assessment including SSEPs (48- 72h after CA) was routinely performed and who had N20PRES. Additional data included clinical examination (pupillary reflexes, motor response, myoclonus), EEG (reactivity to painful stimuli, malignant pattern, i.e. burst-suppression or suppressed background, seizures) during the first 48h and peak serum neuronspecific enolase (NSE) concentrations over the first 72h after CA. Neurological outcome was assessed at 3 months using the Cerebral Performance Categories (3-5= PNO; 1-2= GNO). Results: We studied 532 patients; 389(73%) were N20PRES and 191(49%) of these had a PNO. Compared to patients with a GNO, those with a PNO more frequently had absent pupillary reflexes (18% vs. 2%; p<0.001), absent or extension motor response on day 2-3 (57% vs. 19%; p<0.001), myoclonus (14% vs. 2%;p<0.001), malignant (27% vs. 7%; p<0.001) and non-malignant (22% vs. 5%; p< 0.001)EEG patterns; they also had higher NSE concentrations (27.4 [17.7-62.1] - n=92 vs. 19.0[14.3-29] mcg/L - n=143; p<0.001). Conclusions: In patients with N20PRES, prognostication may be reliably guided by clinical, EEG and biochemical signs reflecting severe post-anoxic cerebral damage. Presence of early malignant EEG patterns, absence of pupillary reflexes, absent or posturing motor response and myoclonus on day 2-3 are associated with PNO. Serum NSE concentrations may also be useful for predicting neurological outcome. These findings support the use of multimodal assessment in these patients.

32. The clinical characteristics of patients with preserved cortical evoked potentials after post-anoxic coma and poor neurological outcome

Authors Nobile L.; Creteur J.; Vincent J.-L.; Taccone F.S.; Goldsztejn N.
Source Anesthesia and Analgesia; Sep 2016; vol. 123 (no. 3); p. 159
Publication Date Sep 2016
Publication Type(s) Conference Abstract
Database EMBASE
Available at Anesthesia & Analgesia from Ovid (LWW Total Access Collection 2015 - Q1 with Neurology)

Abstract Background & Objectives: Bilateral absence of cortical response to somatosensory evoked potentials (SSEPs) after cardiac arrest (CA)is an established marker of poor neurological outcome. However, the presence of these responses ($N20_{PRES}$) has failed to reliably predict good neurological outcome. In the setting of multimodal monitoring, few data are available on the clinical characteristics of $N20_{PRES}$ patients with poor neurological outcome. Materials & Methods: Retrospective analysis of an institutional database (January 2010 to January 2013) including all adult patients admitted to the Intensive Care Unit (ICU) after CA and undergoing SSEPs between 48 and 72 hours after cardiac arrest. All patients underwent targeted temperature management (TTM; 32-34degreeC) for 24 hours. We collected the absence of pupillary reflexes, absent or posturing motor response and status myoclonus on day 2-3 and electroencephalography (EEG) data (e.g. absence of reactivity to painful stimuli; presence of a malignant pattern, such as burst-suppression or flat tracings; electroencephalographic status epilepticus) after return to normothermia. Poor neurological outcome (PNO) was defined as a Cerebral Performance Categories of 3-5 at assessed at 3 months Results: We studied 126 patients, including 81 with $N20_{PRES}$; 55 had PNO. Demographics, comorbidities and CA characteristics were similar between groups except for non-cardiac origin of arrest and non-shockable rhythm which were more frequent in patients with PNO than in the others. PNO Patients with $N20_{PRES}$ were more likely to have absent pupillary reflexes (31% vs. 0%; p=0.003), absent or posturing motor response (51% vs. 11%; p=0.001) on day 2-3 than the other patients. They were also more likely to have a malignant EEG pattern, particularly burst suppression (18% vs. 0%; p=0.02) and a non-reactive EEG (75% vs. 27%; p<0.001). All patients with absent pupillary reflexes on day 2-3 had a PNO (p=0.003). Conclusion: In patients with preserved cortical responses to painful stimuli prognostication may be reliably guided by clinical and EEG signs. The presence of early malignant EEG patterns and the absence of pupillary reflexes on day 2-3 are associated with poor neurological outcome The use of a multimodal assessment may be helpful to guide neuroprognostication in this setting.

33. Neurological prognostication after cardiac arrest and targeted temperature management 33degreeC versus 36degreeC: Results from a randomised controlled clinical trial

Authors Dragancea I.; Cronberg T.; Horn J.; Kuiper M.; Friberg H.; Nielsen N.; Ullen S.; Wetterslev J.; Cranshaw J.; Hassager C.
Source Resuscitation; Aug 2015; vol. 93 ; p. 164-170
Publication Date Aug 2015
Publication Type(s) Article
PubMedID 25921544
Database EMBASE
Abstract Background: The reliability of some methods of neurological prognostication after out-of-hospital cardiac arrest has been questioned since the introduction of induced hypothermia. The aim of this study was to determine whether different treatment temperatures after resuscitation affected the prognostic accuracy of clinical neurological findings and somatosensory evoked potentials (SSEP) in comatose patients. Methods: We calculated sensitivity and false positive rate for Glasgow Coma Scale motor score (GCS M), pupillary and corneal reflexes and SSEP to predict poor neurological outcome using prospective data from the Target Temperature Management after Out-of-Hospital Cardiac Arrest Trial which randomised 939 comatose survivors to treatment at either 33. degreeC or 36. degreeC. Poor outcome was defined as severe disability, vegetative state or death (Cerebral Performance Category scale 3-5) at six months. Results: 313 patients (33%) were prognostically assessed; 168 in the 33. degreeC, and 145 in the 36. degreeC group. A GCS M <=2 had a false positive rate of 19.1% to predict poor outcome due to nine false predictions. Bilaterally absent pupillary reflexes had a false positive rate of 2.1% and absent corneal reflexes had a false positive rate of 2.2% due to one false prediction in each group. The false positive rate for bilaterally absent SSEP N20-peaks was 2.6%. Conclusions: Bilaterally absent pupillary and corneal reflexes and absent SSEP N20-peaks were reliable markers of a poor prognosis after resuscitation from out-of-hospital cardiac arrest but low GCS M score was not. The reliability of the tests was not altered by the treatment temperature.
Copyright © 2015 Elsevier Ireland Ltd.

34. Amplitudes of SSEP and outcome in cardiac arrest survivors

Authors Endisch C.; Ploner C.J.; Leithner C.; Storm C.
Source Neurology; Nov 2015; vol. 85 (no. 20); p. 1752-1760
Publication Date Nov 2015
Publication Type(s) Article
PubMedID 26491086
Database EMBASE

Abstract Objective: To investigate the relationship between somatosensory evoked potential (SSEP) amplitudes and neurologic outcome after cardiac arrest. Methods: We prospectively studied SSEPs, recorded 24 hours to 4 days after cardiac arrest, in patients with targeted temperature management. SSEP amplitude was defined pragmatically as the highest short-latency amplitude of 4 cortical recordings (2 per side, CP3/CP4 vs Fz) at least 4.5 ms after the spinal SSEP. Cerebral performance category (CPC) was determined upon intensive care unit discharge. CPC 1-3 was defined as good, CPC 4-5 as poor outcome. Results: Of 318 patients, 25 had incomplete recordings, no reproducible spinal SSEP, or high noise level. Of the remaining 293 patients, 137 (47%) had poor and 156 (53%) good outcome. The lowest amplitude in a survivor with good outcome was 0.62 mV. All 78 patients with lower amplitudes had poor outcome. None of 27 patients with CPC 4 (unresponsive wakefulness) had amplitudes above 2.5 mV. In the majority of 24 patients who died despite amplitudes above 2.5 mV, clinical course and other prognostic parameters argued against severe hypoxic encephalopathy. Conclusions: The prognostic value of SSEPs extends beyond an absent/present dichotomy. Absent and very low amplitude SSEPs appear to be highly predictive of poor outcome after cardiac arrest. Prospective external validation of the lower threshold found in our study is necessary. SSEP recordings should not be used for prognostication if noise could mask potentials with critically low amplitudes. High SSEP amplitudes argue against severe hypoxic encephalopathy.
Copyright © 2015 American Academy of Neurology.

35. Target temperature management of 33degreeC and 36degreeC in patients with out-of-hospital cardiac arrest with initial non-shockable rhythm - A TTM sub-study

Authors Frydland M.; Kjaergaard J.; Wanscher M.; Winther-Jensen M.; Hassager C.; Erlinge D.; Nielsen N.; Pellis T.; Aneman A.; Friberg H.; Hovdenes J.; Horn J.; Wetterslev J.; Wise M.P.; Kuiper M.; Stammet P.; Cronberg T.; Gasche Y.
Source Resuscitation; 2015; vol. 89 ; p. 142-148
Publication Date 2015
Publication Type(s) Article
Database EMBASE
Abstract Purpose: Despite a lack of randomized trials in comatose survivors of out-of-hospital cardiac arrest (OHCA) with an initial non-shockable rhythm (NSR), guidelines recommend induced hypothermia to be considered in these patients. We assessed the effect on outcome of two levels of induced hypothermia in comatose patient resuscitated from NSR. Methods: Hundred and seventy-eight patients out of 950 in the TTM trial with an initial NSR were randomly assigned to targeted temperature management at either 33 degreeC (TTM33, n= 96) or 36 degreeC (TTM36, n= 82). We assessed mortality, neurologic function (Cerebral Performance Score (CPC) and modified Rankin Scale (mRS)), and organ dysfunction (Sequential Organ Failure Assessment (SOFA) score). Results: Patients with NSR were older, had longer time to ROSC, less frequently had bystander CPR and had higher lactate levels at admission compared to patients with shockable rhythm, p< 0.001 for all. Mortality in patients with NSR was 84% in both temperature groups (unadjusted HR 0.92, adjusted HR 0.75; 95% CI 0.53-1.08, p=. 0.12). In the TTM33 group 3% survived with poor neurological outcome (CPC 3-4, mRS 4-5), compared to 2% in the TTM36 group (adjusted OR 0.67; 95% CI 0.08-4.73, p= 0.69 for both). Thirteen percent in the TTM33 group and 15% in the TTM36 group had good neurologic outcome (CPC 1-2, mRS 0-3, OR 1.5, CI 0.21-12.5, p= 0.69). The SOFA-score did not differ between temperature groups. Conclusion: Comatose patients after OHCA with initial NSR continue to have a poor prognosis. We found no effect of targeted temperature management at 33. degreeC compared to 36 degreeC in these patients.
Copyright © 2015 Elsevier Ireland Ltd.

36. Outcomes of asphyxial cardiac arrest patients who were treated with therapeutic hypothermia: A multicentre retrospective cohort study

Authors Wee J.H.; Park J.H.; Park K.N.; Choi S.P.; You Y.H.; Lim H.; Choi W.J.; Lee B.K.
Source Resuscitation; 2015; vol. 89 ; p. 81-85
Publication Date 2015
Publication Type(s) Article
Database EMBASE

Abstract Introduction: While therapeutic hypothermia (TH) is in clinical use, its efficacy in certain patient groups is unclear. This study was designed to describe the characteristics and outcomes of patients with out-of-hospital cardiac-arrest (OHCA) caused by asphyxia, who were treated with TH. Patients and methods: A multicentre, retrospective, registry-based study was performed using data from the period 2007-2012. Comatose patients who were treated with TH after asphyxial cardiac arrest were included, while those who with cardiac arrest attributed to hanging, drowning or gas intoxication were excluded. Results: Of a total of 932 OHCA patients in the registry, 111 were enrolled in this study. The mean age was 65.8 +/- 16.3 years with individuals who were >=65 years of age accounted for 61.3% of the cohort. Foreign-body airway obstruction was the most common cause (70.3%) of the cardiac arrest. Eighty patients (72.1%) presented with an initial non-shockable rhythm. In all institutions target TH temperatures were 32-34 degreeC, but TH maintenance times varied. A total of 52 patients (46.8%) survived, of whom six patients (5.4%) showed a good neurologic outcome (cerebral performance category scale 1-2). The pupil light reflex, corneal reflex and time to return of spontaneous circulation (p= 0.012, 0.015 and 0.032, respectively) were associated with survival. Witnessed arrest, age, previous lung disease, bystander basic life support and time factors were not associated with survival. Conclusion: About half of patients who underwent TH after asphyxial cardiac arrest survived, but a very small number showed a good neurologic outcome. The TH maintenance times were not uniform in these patients. Additional research regarding both the appropriate TH guidelines for patients with asphyxial cardiac arrest and improvement of their neurologic outcome is needed.
Copyright © 2014 Elsevier Ireland Ltd.

37. The characteristics of patients with bilateral absent evoked potentials after post-anoxic injury

Authors Nobile L.; Goldsztejn N.; Creteur J.; Vincent J.-L.; Taccone F.S.
Source Critical Care Medicine; Dec 2015; vol. 43 (no. 12); p. 44
Publication Date Dec 2015
Publication Type(s) Conference Abstract
Database EMBASE
Available at Critical Care Medicine from Ovid (LWW Total Access Collection 2015 - Q1 with Neurology)
Available at Critical Care Medicine from Ovid (Journals @ Ovid) - London Health Libraries

Abstract Learning Objectives: Bilateral absence of cortical response (N20ABS) to somatosensory evoked potentials (SSEPs) is an established marker of poor neurologic outcome after cardiac arrest (CA). However, few data are available on the clinical characteristics of such patients in comparison to those with present cortical response. Methods: Retrospective analysis of an institutional database (Jan 2010- Janu 2013) including adult patients admitted to the Intensive Care Unit after CA and undergoing SSEPs 48-72 hr after arrest. All patients underwent targeted temperature management (TTM; 32-34degreeC) for 24 hr. We collected the absence of pupillary reflexes; absent or posturing motor response; status myoclonus (on day 2-3); electroencephalography (EEG) data (absence of reactivity to painful stimuli; presence of a malignant pattern, e.g. burst-suppression or flat tracings; status epilepticus) at normothermia. Outcome was assessed at 3 mo using the Cerebral Performance Categories (3-5 = poor recovery; 1-2 = good recovery). Results: We studied 42 patients (N20ABS n=19). Demographics, comorbidities and CA characteristics were similar between groups; 11 patients (26%) had a good neurologic recovery and none of them had N20ABS. Patients with N20ABS had a higher incidence of absent pupillary reflexes (14/19 vs. 4/23; p=0.0004), absent or posturing motor response (18/19 vs. 10/23; p=0.0007) and status myoclonus (4/19 vs. 0/23; p=0.03) on day 2-3 than others. Also, patients with N20ABS presented more frequently a malignant EEG patterns (16/19 vs. 5/23; p=0.0001) and a non-reactive EEG (19/19 vs. 5/23; p<0.0001) than others. The N20ABS was associated with the concomitant presence of EEG findings (e.g. non-reactive EEG or malignant pattern) and clinical signs (e.g. bilateral absence of pupillary reflexes or status myoclonus on day 2-3 after arrest) of poor prognosis in 12/19 of patients when compared to 3/23 in others (p<0.0001). Conclusions: Bilateral absence of N20 to SSEPs is frequently associated with the presence of clinical and EEG signs of poor prognosis. These data support that N20ABS reflect severe post-anoxic injury.

38. Yeast central nervous system infection in a critically ill patient: A case report

Authors Frantzeskaki F.; Diakaki C.; Rizos M.; Theodorakopoulou M.; Papadopoulos P.; Antonopoulou A.; Nikitas N.; Lignos M.; Paramythiotou E.; Armaganidis A.; Dimopoulos G.; Brountzos E.; Velegraki A.; Panagyotides J.
Source Journal of Medical Case Reports; Jul 2014; vol. 8 (no. 1)
Publication Date Jul 2014
Publication Type(s) Article
PubMedID 25026870
Database EMBASE
Available at Journal of Medical Case Reports from BioMed Central
Available at Journal of Medical Case Reports from Europe PubMed Central - Open Access
Available at Journal of Medical Case Reports from PubMed Central

Abstract Introduction. Invasive fungal infections are alarmingly common in intensive care unit patients; invasive fungal infections are associated with increased morbidity and mortality. Risk factors are the increased use of indwelling central venous catheters, the use of broad spectrum antibiotics, parenteral nutrition, renal replacement therapy and immunosuppression. Diagnosis of these infections might be complicated, requiring tissue cultures. In addition, therapy of invasive fungal infections might be difficult, given the rising resistance of fungi to antifungal agents. Case presentation. We describe the case of a 28-year-old Greek man with yeast central nervous system infection. Conclusions: Difficult-to-treat fungal infections may complicate the clinical course of critically ill patients and render their prognosis unfavorable. This report presents a case that was rare and difficult to treat, along with a thorough review of the investigation and treatment of these kinds of fungal infections in critically ill patients. © 2014 Frantzeskaki et al.; licensee BioMed Central Ltd.

39. Clinical examination for prognostication in comatose cardiac arrest patients

Authors Greer D.M.; Scripko P.D.; Cash S.; Yang J.; Sims J.R.; Wu O.; Hafler J.P.; Schoenfeld D.A.; Furie K.L.
Source Resuscitation; Nov 2013; vol. 84 (no. 11); p. 1546-1551
Publication Date Nov 2013
Publication Type(s) Article
PubMedID 23954666
Database EMBASE
Available at Resuscitation from PubMed Central

Abstract Objective: To build new algorithms for prognostication of comatose cardiac arrest patients using clinical examination, and investigate whether therapeutic hypothermia influences the value of the clinical examination. Methods: From 2000 to 2007, 500 consecutive patients in non-traumatic coma were prospectively enrolled, 200 of whom were post-cardiac arrest. Outcome was determined by modified Rankin Scale (mRS) score at 6 months, with mRS. <=. 3 indicating good outcome. The clinical examination was performed on days 0, 1, 3 and 7 post-arrest, and clinical variables analyzed for importance in prognostication of outcome. A classification and regression tree analysis (CART) was used to develop a predictive algorithm. Results: Good outcome was achieved in 9.9% of patients. In CART analysis, motor response was often chosen as a root node, and spontaneous eye movements, pupillary reflexes, eye opening and corneal reflexes were often chosen as splitting nodes. Over 8% of patients with absent or extensor motor response on day 3 achieved a good outcome, as did 2 patients with myoclonic status epilepticus. The odds of achieving a good outcome were lower in patients who suffered asystole (OR 0.187, 95% CI: 0.039-0.875, p= 0.033) compared with ventricular fibrillation or non-perfusing ventricular tachycardia, but some still achieved good outcome. The absence of pupillary and corneal reflexes on day 3 remained highly reliable for predicting poor outcome, regardless of therapeutic hypothermia utilization. Conclusion: The clinical examination remains central to prognostication in comatose cardiac arrest patients in the modern area. Future studies should incorporate the clinical examination along with modern technology for accurate prognostication. © 2013 Elsevier Ireland Ltd.

40. Moderate therapeutic hypothermia in a patient with acute pancreatitis: Case report and review of the literature

Authors Da Silva I.R.F.; McWilliams L.
Source Pancreas; Apr 2013; vol. 42 (no. 3); p. 544-545
Publication Date Apr 2013
Publication Type(s) Letter
PubMedID 23486366
Database EMBASE
Available at Pancreas from Ovid (LWW Total Access Collection 2015 - Q1 with Neurology)

41. Self-fulfilling prophecies through withdrawal of care: Do they exist in traumatic brain injury, too?

Authors Muehlschlegel S.; Izzy S.; Compton R.; Carandang R.; Hall W.
Source Neurocritical Care; Dec 2013; vol. 19 (no. 3); p. 347-363
Publication Date Dec 2013
Publication Type(s) Article
PubMedID 24132565
Database EMBASE
Available at Neurocritical Care from SpringerLink

Abstract Objective: We examined factors associated with withdrawal of care (WOC) in moderate-severe traumatic brain injury (msTBI) patients, and how WOC may affect short-term mortality and receipt of neurosurgery. Variability in msTBI-related outcome prognostication by clinicians from different specialties was also assessed. Methods: Rates of WOC, factors associated with WOC, and the relation between WOC and in-hospital case-fatality rate (CFR) and neurosurgery were determined in 232 prospectively enrolled msTBI patients in the ongoing OPTIMISM Study at a level-1 trauma center. In a concomitant web-based survey with clinical vignettes, outcome prognostication comfort, treatment aggressiveness, and WOC recommendations were examined among 106 respondents from neurology, neurocritical care, neurosurgery, trauma and anesthesia/critical care. Results: The average age of the study sample was 53 years, with a median Glasgow Coma Scale of 6. The in-hospital CFR was 36 and 68 % of patients had WOC. Factors independently associated with WOC were advanced age, pupillary reactivity, lower intensive care unit-length-of-stay, pre- and in-hospital cardiac arrest, herniation, intracranial pressure crisis, and pre-existing endocrine disease. Inclusion of WOC in our multivariable regression model predicting in-hospital CFRs negated all other variables. Survey results suggested that in younger patients, some clinicians prognosticated overly pessimistically based upon data available at the time of presentation. Conclusion: In our msTBI cohort, WOC was the most important predictor of in-hospital mortality. We identified several important independent predictors of WOC. Large within-center variability in msTBI outcome prognostication with varying levels of possible clinical nihilism exists, which may form the basis of self-fulfilling prophecies. © 2013 Springer Science+Business Media New York.

42. Usefulness of a simple prognostication score in prediction of the prognoses of patients with out-of-hospital cardiac arrests

Authors Ishikawa S.; Niwano S.; Imaki R.; Izumi T.; Takeuchi I.; Soma K.; Irie W.; Kurihara K.; Toyooka T.
Source International Heart Journal; 2013; vol. 54 (no. 6); p. 362-370
Publication Date 2013
Publication Type(s) Article
PubMedID 24309445
Database EMBASE

Abstract Sudden cardiac death is a serious problem in public health but the overall survival rate of out-of-hospital cardiac arrests (OHCAs) remains low. In this study, we identif ed clinical parameters to predict the prognosis of OHCA patients and proposed a simple prognostication score for prediction of their prognoses. The study population consisted of 750 consecutive patients with OHCAs of internal cause who were transported to our institute from July 2008 to June 2010. They were divided into survivors and nonsurvivors, and clinical parameters were compared between them to detect sig-nif cant parameters for prediction of their prognoses. The population of those who survived at 1 month numbered 34. Multivariate analysis exhibited 10 independent predictive factors of survival, which included witnessed cardiac arrest and bystander-initiated CPR. When the prognostication score was calculated from these independent predictive factors, a score of >= 6 points indicated survival with a sensitivity of 88.6% and a specif city of 97.6%. When the patients were divided into younger and older populations with a threshold of 70 years, these values were 94.1% and 96.1% in younger but 70.0% and 98.4% in older patients, respectively. In retrospective observation, a simple prognostication score was useful to predict patient prognoses in OHCAs, but its usefulness was limited in an older population.

43. Infrared pupillometry to detect the light reflex during cardiopulmonary resuscitation: A case series

Authors Behrends M.; Niemann C.U.; Larson M.D.
Source Resuscitation; Oct 2012; vol. 83 (no. 10); p. 1223-1228
Publication Date Oct 2012
Publication Type(s) Article
PubMedID 22659054
Database EMBASE

Abstract Background: The presence or absence of the pupillary light reflex following cardiopulmonary resuscitation has been shown to have prognostic value. We asked whether the light reflex could be objectively measured during cardiopulmonary resuscitation in humans and whether the quality of the reflex was associated with outcome. Methods: Sixty-seven in-hospital code blue alerts were attended of which 30 met our inclusion criteria. Portable infrared pupillometry was used to measure the light reflex during each code. The reliability of the presence of the light reflex during each code as a predictor of survival and neurological outcome was analyzed statistically using the Barnard's Exact test. Results: In 25 patients (83%) the pupillary light reflex was detectable throughout or during a part of the resuscitation. Continuous presence of the light reflex or absence for less than 5. min during resuscitation was associated with early survival of the code and a good neurological outcome. In contrast, no patients without a light reflex or with a gradually deteriorating light reflex survived the code and absence of a pupillary light reflex for more than 5. min was associated with an unfavorable outcome. Conclusion: Portable infrared pupillary measurements can reliably demonstrate the presence and quality of the pupillary light reflex after cardiac arrest and during resuscitation. In our limited case series, the presence of the pupillary light reflexes obtained in serial measurements during resuscitation was associated with early survival and a favorable neurological status in the recovery period. © 2012 Elsevier Ireland Ltd.

44. A case of inaccurate prognostication after the ARCTIC protocol

Authors Chen C.J.; Coyne P.J.; Lyckholm L.J.; Smith T.J.
Source Journal of Pain and Symptom Management; Jun 2012; vol. 43 (no. 6); p. 1120-1125
Publication Date Jun 2012
Publication Type(s) Article
PubMedID 22651951
Database EMBASE
Abstract Therapeutic hypothermia (ARCTIC, or Advanced Resuscitation Cooling Therapeutics and Intensive Care protocol) is a widely recommended intervention to improve mortality and neurologic outcomes after cardiac arrest. However, neurologic outcomes are difficult to predict soon after cardiac arrest in the setting of hypothermia, as illustrated by this case report. A 60-year-old man had witnessed cardiac arrest at home. He was defibrillated twice, with return of spontaneous circulation, and cooled to 33degreeC for 24 hours. Neurologic exam on Day 6 revealed limited brainstem reflexes, and the intensive care unit team discussed with the patient's family that his prognosis for neurologic recovery was poor. Palliative care was consulted to participate in a goals-of-care meeting. Just prior to the meeting on Day 7, the patient awoke. He fully recovered and walked out of the hospital on Day 18. Prior to induced hypothermia, indicators of poor outcome included lack of one or more brainstem reflexes (pupillary or corneal reflex), absence of motor response at 72 hours, myoclonus, status epilepticus, electroencephalogram with generalized suppression, and absent bilateral cortical N20 response to somatosensory-evoked potentials. However, several studies have found these indicators to be unreliable after hypothermia. This may be the result of sedatives, which can affect physical examination and electroencephalogram results, and delayed clearance. Because of the unreliability of prognostication tests within the first 72 hours of hypothermic protocols in the setting of sedation, it appears prudent in some cases to delay final prognosis discussions until at least six days postcardiac arrest and after neurologic evaluation is done with patients sedative-free. © 2012 U.S. Cancer Pain Relief Committee. Published by Elsevier Inc. All rights reserved.

45. Utility cranial nerve testing obtained in the emergency department as early predictors of neurologic outcome post cardiac arrest

Authors Kessler J.; Medado P.; Cloyd J.; Wilburn J.; Engle T.; O'Neil B.
Source Annals of Emergency Medicine; Oct 2012; vol. 60 (no. 4)
Publication Date Oct 2012
Publication Type(s) Conference Abstract
Database EMBASE
Available at Annals of Emergency Medicine from Ovid (Journals @ Ovid) - Remote Access
Available at Annals of Emergency Medicine from Ovid (Journals @ Ovid) - London Health Libraries
Abstract Study Objective: Severe neurologic deficit is a major morbidity in patients who survive cardiac arrest. Early prognostication of neurologic function after survival from cardiac arrest in the emergency department (ED) would aid in resource allocation and potentially reduce emotional stress. Lack of cranial nerve findings post-resuscitation is often thought to portend a poor neurologic outcome. Methods: We obtained cranial nerve testing in the ED on adult patients post cardiac arrest with ROSC being admitted to the hospital. Our convenience sample yielded 39 patients and informed consent was obtained from their legal authorized representative. Cranial nerve testing was conducted after initial ROSC in the ED. We collected dichotomous present/absent data on 9 neurological categories: pupillary reflex, corneal reflex, doll's eye, response to pain, myoclonus, seizure activity, spontaneous respirations, cold caloric response and response to verbal stimuli. Neurological outcome was determined using Glasgow-Pittsburgh Outcome Categorization of Brain Injury Cerebral Performance Categories (CPC) at hospital discharge. CPC of 1-2 was dichotomized as a <> outcome and 3-5 as <

46. Predictors of poor neurologic outcome in patients after cardiac arrest treated with hypothermia: A retrospective study

Authors Bisschops L.L.A.; Bons S.; van der Hoeven J.G.; Hoedemaekers C.W.E.; van Alfen N.
Source Resuscitation; Jun 2011; vol. 82 (no. 6); p. 696-701
Publication Date Jun 2011
Publication Type(s) Article
PubMedID 21419561
Database EMBASE

Abstract Introduction: Outcome studies in patients with anoxic-ischemic encephalopathy focus on the early and reliable prediction of an outcome no better than a vegetative state or severe disability. We determined the effect of mild therapeutic hypothermia on the validity of the currently used clinical practice parameters. Methods: We conducted a retrospective cohort study of adult comatose patients after cardiac arrest treated with hypothermia. All data were collected from medical charts and laboratory files and analyzed from the day of admission to the intensive care unit until day 7, discharge from the intensive care unit or death using the Utstein definitions for the registration of the data. Results: We analyzed the data of 103 patients. The combination of an M1 or M2 on the Glasgow Coma Scale or absent pupillary reactions or absent corneal reflexes on day 3 was present in 80.6% of patients with an unfavourable and 11.1% of patients with a favourable outcome. The combination of M1 or M2 and absent pupillary reactions to light and absent corneal reflexes on day 3 was present in 14.9% of patients with an unfavourable and none of the patients with a favourable outcome. None of the patients with a favourable outcome had a bilaterally absent somatosensory evoked potential of the median nerve. The value of electroencephalogram patterns in predicting outcome was low, except for reactivity to noxious stimuli. Conclusions: No single clinical or electrophysiological parameter has sufficient accuracy to determine prognosis and decision making in patients after cardiac arrest, treated with hypothermia. © 2011 Elsevier Ireland Ltd.

47. Atropine, fixed dilated pupils and prognostication following cardiac arrest

Authors MacDougall-Davis S.
Source Resuscitation; Feb 2011; vol. 82 (no. 2); p. 232
Publication Date Feb 2011
Publication Type(s) Letter
PubMedID 21145158
Database EMBASE

48. Sedation confounds outcome prediction in cardiac arrest survivors treated with hypothermia

Authors Samaniego E.A.; Mlynash M.; Caulfield A.F.; Eyngorn I.; Wijman C.A.C.
Source Neurocritical Care; Aug 2011; vol. 15 (no. 1); p. 113-119
Publication Date Aug 2011
Publication Type(s) Article
PubMedID 20680517
Database EMBASE
Available at Neurocritical Care from SpringerLink
Available at Neurocritical Care from PubMed Central

Abstract Background Therapeutic hypothermia is commonly used in comatose survivors' post-cardiopulmonary resuscitation (CPR). It is unknown whether outcome predictors perform accurately after hypothermia treatment. Methods Post-CPR comatose survivors were prospectively enrolled. Six outcome predictors [pupillary and corneal reflexes, motor response to pain, and somatosensory- evoked potentials (SSEP) >72 h; status myoclonus, and serum neuron-specific enolase (NSE) levels <72 h] were systematically recorded. Poor outcome was defined as death or vegetative state at 3 months. Patients were considered "sedated" if they received any sedative drugs <=12 h prior the 72 h neurological assessment. Results Of 85 prospectively enrolled patients, 53 (62%) underwent hypothermia. Furthermore, 53 of the 85 patients (62%) had a poor outcome. Baseline characteristics did not differ between the hypothermia and normothermia groups. Sedative drugs at 72 h were used in 62 (73%) patients overall, and more frequently in hypothermia than in normothermia patients: 83 versus 60% (P = 0.02). Status myoclonus <72 h, absent cortical responses by SSEPs >72 h, and absent pupillary reflexes >72 h predicted poor outcome with a 100% specificity both in hypothermia and normothermia patients. In contrast, absent corneal reflexes >72 h, motor response extensor or absent >72 h, and peak NSE >33 ng/ml <72 h predicted poor outcome with 100% specificity only in non-sedated patients, irrespective of prior treatment with hypothermia. Conclusions Sedative medications are commonly used in proximity of the 72-h neurological examination in comatose CPR survivors and are an important prognostication confounder. Patients treated with hypothermia are more likely to receive sedation than those who are not treated with hypothermia. © Springer Science+Business Media, LLC 2010.

49. Prognostic value of brain diffusion- after weighted imaging cardiac arrest

Authors Wijman C.A.C.; Mlynash M.; Caulfield A.F.; Hsia A.W.; Eyngorn I.; Albers G.W.; Bammer R.; Moseley M.; Fischbein N.
Source Annals of Neurology; Apr 2009; vol. 65 (no. 4); p. 394-402
Publication Date Apr 2009
Publication Type(s) Article
PubMedID 19399889
Database EMBASE
Available at Annals of Neurology from Wiley Online Library Science , Technology and Medicine Collection 2017
Available at Annals of Neurology from PubMed Central

Abstract Objective: Outcome prediction is challenging in comatose postcardiac arrest survivors. We assessed the feasibility and prognostic utility of brain diffusion-weighted magnetic resonance imaging (DWI) during the first week. Methods: Consecutive comatose postcardiac arrest patients were prospectively enrolled. AWI data of patients who met predefined specific prognostic criteria were used to determine distinguishing apparent diffusion coefficient (ADC) thresholds. Group 1 criteria were death at 6 months and absent motor response or absent pupillary reflexes or bilateral absent cortical responses at 72 hours or vegetative at 1 month. Group 2 criterion was survival at 6 months with a Glasgow Outcome Scale score of 4 or 5 (group 2A) or 3 (group 2B). The percentage of voxels below different ADC thresholds was calculated at 50 x 10^{-6} mm^2/sec intervals. Results: Overall, 86% of patients underwent DWI. Fifty-one patients with 62 brain DWIs were included. Forty patients met the specific prognostic criteria. The percentage of brain volume with an ADC value less than 650 to 700 x $10^{-6}mm^2$/sec best differentiated between Group 1 and Groups 2A and 2B combined ($p < 0.001$), whereas the 400 to 450 x 10^{-6} mm^2/sec threshold best differentiated between Groups 2A and 2B ($p = 0.003$). The ideal time window for prognostication using DWI was between 49 and 108 hours after the arrest. When comparing DWI in this time window with the 72-hour neurological examination, DWI improved the sensitivity for predicting poor outcome by 38% while maintaining 100% specificity ($p =0.021$). Interpretation: Quantitative DWI in comatose postcardiac arrest survivors holds promise as a prognostic adjunct. © 2009 American Neurological Association.

50. Neurologic prognosis after cardiac arrest

Authors Young G.B.
Source New England Journal of Medicine; Aug 2009; vol. 361 (no. 6); p. 605-611
Publication Date Aug 2009
Publication Type(s) Article
PubMedID 19657124
Database EMBASE
Available at The New Englang Journal of Medicine from Massachusetts Medical Society
Available at The New Englang Journal of Medicine from ProQuest (Hospital Premium Collection) - NHS Version

Abstract A 55-year-old man collapses while jogging through the park. A bystander finds him unconscious and without a pulse and initiates cardiopulmonary resuscitation (CPR) while an ambulance is summoned. On arrival in the emergency room, the patient is in ventricular fibrillation; the partial pressure of oxygen in arterial blood is 200 mm Hg, the pH is 7.25, and the bicarbonate level is 18 mmol per liter. Spontaneous circulation is reestablished, but he remains comatose with absent pupillary reflexes. He is then treated with hypothermia, achieving a core temperature of 34degreeC in 4 hours, which is maintained for 24 hours, after which he remains unconscious. What would you advise regarding his neurologic prognosis? Copyright © 2009 Massachusetts Medical Society.

51. Prognosis and prognostication after cardiac arrest and hypothermia; Results of PROPACII,, a Dutch multicenter, prospective cohort study

Authors Horn J.; Bouwes A.; Binnekade J.; Koelman H.; Hijdra A.; Kuiper M.; Bosch F.; Zandstra D.; Toornvliet A.; Moeniralam H.; Kors B.; Verbeek M.; Weinstein H.
Source Neurocritical Care; Sep 2010; vol. 13
Publication Date Sep 2010
Publication Type(s) Conference Abstract
Database EMBASE
Available at Neurocritical care from SpringerLink

Abstract Introduction: Guidelines for determination of prognosis in patients with post anoxic coma after cardiac arrest are all based on data collected in patients who were not treated with hypothermia. In most hospitals however, this treatment has become standard care. Therefore data regarding the reliability of diagnostic tools after hypothermia used to determine prognosis are needed. The objective of the PROPAC II study was to establish the validity of diagnostic methods to predict poor outcome in patients treated with hypothermia after CPR. Methods: This multicenter prospective cohort study included adult comatose patients admitted after CPR and treated with induced mild hypothermia (32-34degreeC). Data collected: Age, gender, presenting rhythm, time to return of spontaneous circulation, characteristics about hypothermia treatment, serum NSE levels on admission, 12 hrs after reaching target temperature, 36 and 48 hours after CPR, results of SSEP recorded if patients remained in coma after rewarming and wearing off of sedative drugs. GCS and brain stem reflexes were tested 48 and 72 hours after CPR. Neurological outcome was assessed six months after admission with the Glasgow Outcome Scale. Poor outcome was defined as death, vegetative state or severe disability. For SSEP, GCS-M score (M score dichotomy M1-2 versus M3-6), pupillary reaction, corneal reflex and NSE levels >33microg/L, false positive rates (FPR, 1 - specificity) for prediction of poor outcome was calculated with 95% CI. Results: 391 patients were included in 10 collaborating hospitals between September 2007 and October 2009. Patient characteristics: median age 65, 73% male, median time to ROSC 15 min., initial rhythm VF/VT in 77%. Outcome: 205 (53%) patients died, of whom 149 died in the first week after admission. No patient remained in a vegetative state, 9 patients were severely disabled, 49 moderately disabled and 124 made a good recovery. Predicting poor outcome: * SSEP (normothermia) FPR 0.01, 95%CI 0-0.05, * GCS-M score (72 hrs) FPR 0.12, 95%CI 0.07-0.18 * pupillary reaction (72 hrs) FPR 0.01, 95%CI 0-0.07 * Corneal reflex (72 hrs) FPR 0.04 95%CI 0.01-0.13 * NSE (48hrs) FPR 0.07 95%CI 0.04-0-12 Conclusions: This study shows that absent pupillary reaction 72 hours after CPR and absent cortical response in SSEP after rewarming reliably predict poor outcome at six months in patients who remain comatose after CPR and treatment with hypothermia. These tests can be used already shortly after rewarming and wearing off of sedative drugs. The results are in accordance with the recently published results of Rosetti, found in a smaller group of patients from one centre. Further results of analyses will be presented on the conference.

52. Coma anoxico-isquemico en 46 pacientes. Evolucion a treinta dias y su relacion con los reflejos de troncoThe absence of brainstem reflexes does not predict short term mortality in anoxic ischemic coma

Authors Lay-Son R L.; Varas F P.
Source Revista Medica de Chile; Apr 2006; vol. 134 (no. 4); p. 441-446
Publication Date Apr 2006
Publication Type(s) Article
PubMedID 16758079
Database EMBASE
Abstract Background: Anoxic-ischemic coma has a poor outcome with a high rate of mortality and morbidity. Therefore, clinical predictors of prognosis are needed for therapeutic decision-making. Patients and methods: Prospective analysis of 46 patients, 31 male, age range 19-85 years, with anoxic-ischemic coma following cardiac arrest. All the patients included in our study remained comatose with a Glasgow Coma Scale (GCS) score of six or less points, after their stabilization in the Intensive Care Unit. They were evaluated clinically using the pupillary light reflex, corneal reflex and vestibulo-ocular reflex testing, induced by caloric stimulation with cold water. Survival was evaluated using life tables. All patients were followed until the thirtieth day after the anoxic-ischemic event. Results: Thirty five patients (76%) died within the next twenty-nine days, 8 patients (18%) reached the vegetative state, 2 patients (4%) achieved a recovery with disability, and only 1 patient (2%) was discharged without sequelae. One day, five and 30 days survival rates were 89, 53 and 29%, respectively. The abolition of all brainstem reflexes was not a predictor of mortality. Conclusion: Thirty day survival in this group of patients was 29% and the absence of brainstem reflexes was not a predictor of mortality.

53. Coma in the intensive care unit: Predicting awakening following cardiac and respiratory arrest

Authors Tirschwell D.L.
Source CONTINUUM Lifelong Learning in Neurology; Feb 2006; vol. 12 (no. 1); p. 46-69
Publication Date Feb 2006
Publication Type(s) Article
Database EMBASE

Abstract Accurately predicting the chances of awakening following cardiorespiratory arrest is important to help families make decisions about continued medical care. Falsely pessimistic predictions of never awakening (when awakening may have occurred if the patient was supported long enough) should be avoided. The vast majority (approximately 90%) of patients who will awaken do so within the first 3 days, and most patients should be supported for this duration. Absent motor response to pain or absent pupillary light reflex 3 days after cardiac arrest are strongly associated with never awakening. Early myoclonus frequently, but not uniformly, portends an ominous prognosis. Absent bilateral short-latency somatosensory evoked potentials may be the most reliable prognostic test available today, but the sensitivity for identifying patients who will not awaken runs only approximately 44%. Depending on local availability, electroencephalography and biochemical tests may provide supporting information. Imaging results, even with the use of modern magnetic resonance imaging techniques, have not been well-enough characterized in large blinded studies to be useful in predicting outcome. Copyright © Lippincott Williams & Wilkins.

54. Anoxic-ischemic encephalopathy: Clinical and electrophysiological associations with outcome

Authors Young G.B.; Doig G.; Ragazzoni A.
Source Neurocritical Care; Apr 2005; vol. 2 (no. 2); p. 159-164
Publication Date Apr 2005
Publication Type(s) Article
PubMedID 16159058
Database EMBASE
Available at Neurocritical care from SpringerLink

Abstract Introduction: Prognostic determination of patients in coma after resuscitation from cardiac arrest is both common and difficult. We explored clinical and electrophysiological testing to determine their associations with favorable and poor outcomes. Methods: We studied 75 comatose patients resuscitated from cardiac arrest, excluding those who were brain dead or continuously sedated; none received hypothermia therapy. Clinical examinations were performed on day 1. Results: The following proportions recovered awareness: 2 of 18 patients with absent pupillary reflexes; 18 of 57 with preserved pupillary reflexes ($p = 0.08$); 2 of 32 with absent corneal reflexes; 16 of 43 with preserved corneal reflexes ($p = 0.001$); 0 of 15 with absent oculovestibular reflexes; and 8 of 29 with preserved oculovestibular reflexes ($p < 0.037$). Purposeful movements were associated with a high probability of recovery, whereas other categories were unfavorable. Other categories of motor response were associated with an increased proportion of those who died without recovering awareness, but each category had some survivors. Somatosensory evoked potentials (SSEPs) were recorded from 47 patients. One of 21 patients with loss of the N20 component survived, compared with survival of 11 of 26 patients in whom it was present ($p = 0.003$). All 5 patients with preserved N70 responses recovered awareness in a subgroup of 33 patients. Sixteen of 22 subjects with mild electroencephalogram (EEG) abnormalities recovered consciousness, compared with the survival of 3 of 50 patients with malignant EEG patterns ($p = 0.0000001$). Combining SSEP with EEG findings produced even greater predictive value. Conclusion: It seems unlikely that any single test will prove to have 100% predictive value for outcome; further studies combining clinical, EEG, and SSEP testing are warranted. Copyright © 2005 Humana Press Inc. All rights of any nature whatsoever are reserved.

55. Early prognostic criteria for neurological recovery and long-term survival after cardiac arrest

Authors Monasterio F.; Blancas R.; Martin C.; Montans M.
Source Clinical Intensive Care; 1997; vol. 8 (no. 3); p. 126-131
Publication Date 1997
Publication Type(s) Article
Database EMBASE

Abstract Objectives: To search for early prognostic criteria for neurological recovery and to assess long-term survival after an episode of cardiac arrest (CA). Design: A study of clinical prediction tools. Setting: Intensive care unit of a 700-bed university hospital. Subjects: The study was divided into two phases. In Phase I (study group, 1978-88) prognostic criteria were investigated in 144 consecutive patients surviving for more than 24 hours after an episode of CA. In Phase II (validation group, 1989-94) these criteria were validated in a population of 119 patients with similar characteristics. Measurements and main results: Phase I (study group): 56 variables (demographic, clinical and biochemical) were examined as possible predictors of neurological outcome. The following neurological signs - eye opening, pupils, eye movement, oculocephalic and oculovestibular reflexes were grouped as signs of neurological activity (SNA) and were considered abnormal when any one sign was abnormal. The Glasgow coma score (GCS) was also evaluated. The patients were classified into two groups at the end of hospitalisation and at four years: recovered (total recovery or minimal damage) and non-recovered (conscious with serious damage or comatose). Variables were analysed by univariate and multivariate logistical regression analysis, and the positive predictive value (PPV) and the negative predictive value (NPV) of non-recovery were calculated. The survival study was carried out with the Kaplan-Meier method. A value of $p <= 0.05$ was considered statistically significant. The main results were: i) variables predictive of non-recovery: abnormal SNA and a score of GCS < 9, on the third day after the CA (PPV of non-recovery 97% and 95% respectively); ii) good neurological outcome was associated with the duration of the CA and cardiopulmonary resuscitation (CPR) < 5 minutes ($p < 0.001$), and with CA in acute myocardial infarction ($p < 0.01$). Sixty-one per cent of patients who left hospital were in a good clinical condition at four years. Phase II (validation group): with the aim of validating the prognostic criteria found in the previous phase, the SNA and GCS scores on the third day after CA were studied. The non-recovery PPV of these variables was 94% and 95% respectively, similar to the Phase I group. Conclusions: Both a GCS < 9 and an abnormal SNA on the third day after CA are prognostic criteria of poor neurological outcome. The majority of patients who survive and leave hospital after an episode of CA have prolonged survival in good clinical condition.

56. Cardiopulmonary resuscitation: A retrospective review

Authors Denton R.; Thomas A.N.
Source Anaesthesia; 1997; vol. 52 (no. 4); p. 324-327
Publication Date 1997
Publication Type(s) Article
PubMedID 9135182
Database EMBASE
Available at Anaesthesia from Wiley Online Library Free Content - NHS
Available at Anaesthesia from Wiley Online Library Science , Technology and Medicine Collection 2017

Abstract The outcome of patients admitted to intensive care after a cardiac arrest was determined by reviewing intensive care unit records at four hospitals for 1993 and 1994. Of the 112 patients identified, 49 survived intensive care of whom 28 were discharged from hospital. In January 1996, 26 of the 28 patients could be traced; 22 of these were still alive. Seven factors were significantly different between survivors and nonsurvivors. At the cardiac arrest these were the number of direct current shocks ($p < 0.05$) and adrenaline doses ($p < 0.01$) given. In intensive care the factors were the presence of reactive pupils ($p < 0.01$), Glasgow Coma Score ($p < 0.001$), APACHE II score ($p < 0.05$), arterial standard bicarbonate ($p < 0.05$) and the use of inotropes ($p < 0.05$). It was not possible to use individual variables to predict outcome at the time of intensive care unit admission. The results suggest that neurological function is an important determinant of outcome and more sensitive neurophysiological testing might be a useful prognostic tool.

57. [Multi-recurrent cardiac arrest: when to discontinue cardiopulmonary resuscitation?].

Authors Perpoint, T; Peillon, D; Chambost, M; David, G; Royon, O; Combe, C
Source Annales francaises d'anesthesie et de reanimation; Mar 2000; vol. 19 (no. 3); p. 195-197
Publication Date Mar 2000
Publication Type(s) Case Reports Journal Article
PubMedID 10782244
Database Medline

Abstract A 52-year-old man developed an out-of-hospital cardiac arrest complicating a myocardial infarction. After prolonged cardiopulmonary resuscitation, he was admitted to an intensive care unit, where 25 episodes of cardiac arrests occurred within a few hours. Finally the outcome was favourable. This case raises the question of the duration a cardiopulmonary resuscitation in case of out-of-hospital and in-hospital cardiac arrest. The question is to determine how long resuscitation efforts must be prolonged after recurrent cardiac arrests.

58. Predictors for good cerebral performance among adult survivors of out-of-hospital cardiac arrest.

Authors Abe, Toshikazu; Tokuda, Yasuharu; Ishimatsu, Shinichi; SOS-KANTO study group
Source Resuscitation; Apr 2009; vol. 80 (no. 4); p. 431-436
Publication Date Apr 2009
Publication Type(s) Journal Article

PubMedID 19185409
Database Medline
Abstract BACKGROUNDComplete neurological recovery is of great importance to survivors of cardiac arrest. Few studies have explored predictors of good cerebral performance outcomes among these.METHODSWe analyzed data from the SOS-KANTO study, a prospective, multi-center, observational study on patients who had out-of-hospital cardiac arrest. We included patients with Glasgow-Pittsburgh cerebral performance categories (GP-CPC) 1 (good cerebral performance) and 2 (moderate cerebral disability) at 30 days after cardiac arrest.RESULTSAmong 122 eligible patients, 85 (70%) with GP-CPC 1 and 37 (30%) with GP-CPC 2 outcomes were analyzed. More patients with GP-CPC 1 outcome (27%) received conventional cardiopulmonary resuscitation (CPR) than those with GP-CPC 2 outcome (5%). Proportions for receiving cardiac-only resuscitation were not different between the two groups. Based on a multiple logistic-regression model constructed using age and significant variables from bivariate analyses, significant factors for GP-CPC 1 outcome included: conventional bystander CPR compared to no bystander resuscitation with an odds ratio of 5.7 (95% CI, 1.1-30.4); positive pupillary reflex at the time of ED arrival with an odds ratio of 13.7 (95% CI, 3.5-53.7); spontaneous respiration at ED arrival with an odds ratio of 5.98 (95% CI, 1.6-23.0); and cardiac cause of initial arrest with an odds ratio of 5.9 (95% CI, 1.4-25.0).CONCLUSIONSSurvivors of out-of-hospital cardiac arrest with recovery to good cerebral performance were more likely to have cardiac cause of arrest and show positive pupillary reflex and spontaneous respiration at ED arrival.

59. Physiologic predictors of survival in post-traumatic arrest.

Authors Cera, Susan M; Mostafa, Gamal; Sing, Ronald F; Sarafin, Jennifer L; Matthews, Brent D; Heniford, B Todd
Source The American surgeon; Feb 2003; vol. 69 (no. 2); p. 140-144
Publication Date Feb 2003
Publication Type(s) Journal Article
PubMedID 12641355
Database Medline
Available at The American surgeon from ProQuest (Hospital Premium Collection) - NHS Version
Abstract Traumatic cardiac or pulmonary arrest is often associated with a dismal outcome and is considered by many to be an example of medical futility and inappropriate use of resources. This study aimed to identify the predictors of survival in patients experiencing traumatic cardiac arrest. We retrospectively reviewed all trauma patients undergoing cardiopulmonary resuscitation on arrival to the Emergency Department (ED) at an American College of Surgeons-designated Level I trauma center over 4 years. ED survival, hospital survival, and neurologic outcomes on discharge were the primary outcomes. Survival rates were examined in relation to demographics, mechanism of injury, airway management, cardiac electrical rhythm, and pupil size and reactivity. Statistical analyses used chi-square and t tests, P < 0.05 was considered significant. A total of 195 patients arrived in the ED with traumatic cardiac arrest; 34 were pronounced dead on arrival (no signs of life), and no resuscitation efforts were initiated. Of the remaining 161 patients 53 (33%) survived to leave the ED, and only 15 (9%) left the hospital alive. Demographic features were similar in survivors and nonsurvivors. The setting of intubation (prehospital vs ED) did not influence survival (P = 0.36). Penetrating trauma adversely affected survival in the ED (P = 0.01); however, this only approached significance in the final outcome of hospital survival (P = 0.06). The presence of sinus rhythm and nondilated reactive pupils was highly significant in predicting ED and hospital survival (P = 0.001). No patient with agonal rhythm or ventricular fibrillation/tachycardia survived, and 14 of the 15 hospital survivors had reactive pupils on arrival to the ED. We conclude that sinus rhythm and pupil size and reactivity are important physiologic variables that predict potential survival and may be used to guide continuation of resuscitative efforts in patients with traumatic cardiac arrest.

60. Survey of brain temperature management in patients with traumatic brain injury in the Japan neurotrauma data bank.

Authors Suehiro, Eiichi; Koizumi, Hiroyasu; Kunitsugu, Ichiro; Fujisawa, Hirosuke; Suzuki, Michiyasu
Source Journal of neurotrauma; Feb 2014; vol. 31 (no. 4); p. 315-320
Publication Date Feb 2014
Publication Type(s) Journal Article
PubMedID 24047191
Database Medline
Available at Journal of neurotrauma from ProQuest (Hospital Premium Collection) - NHS Version

Abstract The goal of this study was to evaluate the clinical characteristics and effects of brain temperature management in patients with severe traumatic brain injury (TBI). A total of 1091 patients were registered from the Japan Neurotrauma Data Bank Project 2009. Those with a Glasgow Coma Scale (GCS) score of 9 or more, a GCS score of 3, bilateral dilated pupils, or cardiopulmonary arrest on arrival were excluded. This left a total of 401 patients. Patients were classified into three groups: no temperature management, with no intervention for brain temperature (225 patients, 56.1%), intensive normothermia (129 patients, 32.2%), and hypothermia (47 patients, 11.7%). Patient age, GCS score, pupillary abnormality, Injury Severity Score (ISS), intracranial pressure (ICP) monitoring, and outcome according to CT classification (Traumatic Coma Data Bank classification) on admission were examined. Patients were significantly older in the no temperature management group (average age 61.5 years) compared with normothermia (53.6 years) and hypothermia (46.9 years). ICP monitoring was significantly decreased in 85.1% of patients with hypothermia, 42.6% with normothermia, and 14.7% in no temperature management group. Favorable outcome rate was significantly higher with hypothermia (52.4%) compared with normothermia (26.9%) and no temperature management (20.7%) with evacuated mass lesions in contrast to diffuse injury. Multivariate analysis in patients with evacuated mass lesions showed that GCS (≥6 pts), and hypothermia were independent factors related to a favorable outcome. Appropriate thermoregulation of the brain for individual patients with various types of TBI are important.

61. Neurologic prognosis after cardiopulmonary arrest: IV. Brainstem reflexes.

Authors Snyder, B D; Gumnit, R J; Leppik, I E; Hauser, W A; Loewenson, R B; Ramirez-Lassepas, M
Source Neurology; Sep 1981; vol. 31 (no. 9); p. 1092-1097
Publication Date Sep 1981
Publication Type(s) Research Support, Non-u.s. Gov't Journal Article
PubMedID 7196528
Database Medline

62. Prediction of awakening after out-of-hospital cardiac arrest.

Authors Longstreth, W T; Diehr, P; Inui, T S
Source The New England journal of medicine; Jun 1983; vol. 308 (no. 23); p. 1378-1382
Publication Date Jun 1983
Publication Type(s) Research Support, Non-u.s. Gov't Journal Article Research Support, U.s. Gov't, P.h.s.
PubMedID 6843631
Database Medline
Available at The New England journal of medicine from Massachusetts Medical Society
Available at The New England journal of medicine from ProQuest (Hospital Premium Collection) - NHS Version

Abstract To develop a model that would forecast neurologic recovery after out-of-hospital cardiac arrest, we reviewed charts on 389 consecutive patients who were not awake on admission to the hospital after resuscitation from asystole or ventricular fibrillation. The outcome variable was "awakening," which was defined as having comprehensible speech or the ability to follow commands. Predictor variables that we considered included both preadmission and admission data. Using discriminant analysis, we derived models from a 60 per cent random sample of cases and tested the models on the remaining 40 per cent. We judged that the best model contained four variables from the admission examination: motor response, pupillary light response, spontaneous eye movements, and blood glucose (levels below 300 mg per deciliter predicted awakening). Overall correct classification was 80 per cent in the derivation sample and 77 per cent in the test sample. In a simplified form, the model's predictions of awakening had a sensitivity of 0.92, a specificity of 0.65, a positive predictive value of 0.80, and a negative predictive value of 0.84. This rule should be clinically useful in estimating the neurologic prognosis of patients resuscitated after out-of-hospital cardiac arrest.

Strategy 406474

#	Database	Search term	Results
1	Medline	(prognostic factors OR prognostication OR Prognosis OR Prognostic OR Prognostic estimate).ti,ab	492771
2	Medline	PROGNOSIS/ OR "DISEASE-FREE SURVIVAL"/ OR "MEDICAL FUTILITY"/ OR NOMOGRAMS/ OR "TREATMENT OUTCOME"/	1258436
3	Medline	(1 OR 2)	1505520
4	Medline	(asystole OR cardiac arrest OR cardiopulmonary arrest OR circulatory arrest OR sudden cardiac arrest OR SCA OR sudden cardiac death OR SCD).ti,ab	50079
5	Medline	exp "HEART ARREST"/	42190
6	Medline	(4 OR 5)	77821
7	Medline	(3 AND 6)	13343
8	Medline	(infrared pupillometry OR pupillometry OR pupil measurement OR pupil response).ti,ab	2675
9	Medline	(pupillometry).ti,ab	662
10	Medline	(infrared).ti,ab	107020
11	Medline	(9 AND 10)	127
12	Medline	DILATATION/ OR "REFLEX, PUPILLARY"/	12642
13	Medline	(8 OR 11 OR 12)	14799
14	Medline	(7 AND 13)	28
15	EMBASE	(prognostic factors OR prognostication OR Prognosis OR Prognostic OR Prognostic estimate).ti,ab	740205
16	EMBASE	PROGNOSIS/ OR "PROGNOSTIC ASSESSMENT"/	538032
17	EMBASE	(15 OR 16)	922495
18	EMBASE	(asystole OR cardiac arrest OR cardiopulmonary arrest OR circulatory arrest OR sudden cardiac arrest OR SCA OR sudden cardiac death OR SCD).ti,ab	95036
19	EMBASE	exp "HEART ARREST"/	78348
20	EMBASE	(18 OR 19)	124875

21	EMBASE	(17 AND 20)	10167
22	EMBASE	(infrared pupillometry OR pupillometry OR pupil measurement OR pupil response).ti,ab	1216
25	EMBASE	(pupillometry).ti,ab	892
26	EMBASE	(infrared).ti,ab	104173
27	EMBASE	(25 AND 26)	175
28	EMBASE	PUPILLOMETRY/ OR "PUPIL REFLEX"/ OR "PUPIL SIZE"/ OR "PUPILLARY LIGHT REFLEX"/ OR "PUPILLARY REACTION"/	14182
29	EMBASE	(22 OR 27 OR 28)	14329
30	EMBASE	(21 AND 29)	107
31	CINAHL	(prognostic factors OR prognostication OR Prognosis OR Prognostic OR Prognostic estimate).ti,ab	69393
32	CINAHL	PROGNOSIS/ OR "PREDICTIVE VALUE OF TESTS"/ OR WAVEFORMS/	95233
33	CINAHL	(31 OR 32)	141084
34	CINAHL	(asystole OR cardiac arrest OR cardiopulmonary arrest OR circulatory arrest OR sudden cardiac arrest OR SCA OR sudden cardiac death OR SCD).ti,ab	17970
35	CINAHL	exp "HEART ARREST"/	15787
36	CINAHL	(34 OR 35)	25604
37	CINAHL	(33 AND 36)	2399
38	CINAHL	(infrared pupillometry OR pupillometry OR pupil measurement OR pupil response).ti,ab	554
41	CINAHL	(pupillometry).ti,ab	108
42	CINAHL	(infrared).ti,ab	4354
43	CINAHL	(41 AND 42)	14
44	CINAHL	"REFLEX, PUPILLARY"/	276
45	CINAHL	(38 OR 43 OR 44)	759
46	CINAHL	(37 AND 45)	16

47	EMBASE	30 [DT 1990-2018] [Publication types Article OR Journal OR Review] [Human age groups Adult 18 to 64 years OR Aged 65+ years]	47
48	Medline	14 [DT 1990-2018] [Human age groups Adult OR Middle Aged OR Aged OR Aged,80 and over]	14
