# Supplementary material for: Infrared pupillometry to help predict neurological outcome for patients achieving return of spontaneous circulation following cardiac arrest: a systematic review protocol
Source: Syst Rev. 2019 Nov 25;8:286. doi: 10.1186/s13643-019-1209-z (PMC6876106; doi:10.1186/s13643-019-1209-z)
Supplement: Supplementary file 4 — Additional file 4. Cochrane public health group data extraction and assessment template. [file 13643_2019_1209_MOESM4_ESM.docx]

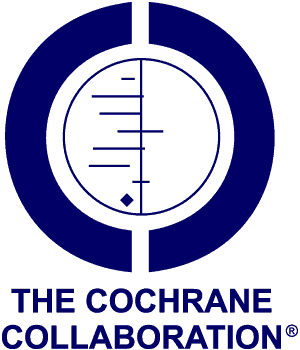


**The Cochrane**

**Public Health Group**

***Data Extraction and Assessment Template***

This form suggests elements which should be addressed in your review and is to be modified in keeping with the following instructions. Some questions may be changed from open-ended questions to specific data items where appropriate. Refer to the Cochrane Handbook when undertaking modifications to this form.

Sections can be expanded and irrelevant sections can be removed. It is difficult to design a single form that meets the needs of all reviews. It is therefore important that you consider your needs carefully prior to data extraction and pilot your process. Elements within the template are not intended for use as a scoring system. The components of the *Risk of Bias Table* have been incorporated into this form. Criteria for judging risk of bias as well as examples of appropriate methods of addressing each form of bias are provided in Chapter 8 of the Cochrane Handbook, particularly Table 8.5.c. For tips on how to enter data into RevMan 5, see “Risk of Bias” tables in the RevMan User Guide. If you are using an additional quality assessment tool you will need to add appropriate questions to reflect the additional components.

*Notes on using a data extraction form:*

- Pilot the Data Extraction Form you plan on using (and note in your protocol that it will, or has, been piloted)
- Be consistent in the order and style you use to describe the information. This will make it easier to complete the Table of Included Studies, prevent you from overlooking information and make reading of the review easier.
- Highlight any missing information as unclear or not described, to make it clear to the reader of your review that the information was not included in the description of the study, not that you forgot to extract it.
- You should include instructions and decision rules on the data collection form. It is crucial that you practice using the form and receive, or give, training if the form was designed by someone other than the person using it.

**Cochrane Public Health Group Data Extraction and Assessment Template (*modify* to suit your review)**

| **Study ID:** | **Report ID :** | Date form completed: |
| --- | --- | --- |
| First author: | Year of study: | Data extractor: |
| Citation: | | |

**1. General Information**

| Publication type Journal Article ⬜ Abstract ⬜ Other (specify e.g. book chapter)___________________ | |
| --- | --- |
| Country of study: | |
| Funding source of study: | Potential conflict of interest from funding? Y / N / unclear |

**2. Study Eligibility**

| **Study Characteristics** | | | | **Page/ Para/ Figure #** |
| --- | --- | --- | --- | --- |
| **Type of study**  (Review authors to add/remove designs based on criteria specified in protocol) | ⬜ Randomised Controlled Trial (RCT)  ⬜ Cluster Randomised Controlled Trial (cluster RCT) | | ⬜ Controlled Before and After (CBA) study   - Contemporaneous data collection - Comparable control site - At least 2 x intervention and 2 x control clusters |  |
|  | ⬜ Interrupted Time Series (ITS)   - At least 3 time points before and 3 after the intervention - Clearly defined intervention point | | ⬜ Other design (specify): |  |
|  | ⬜ A process evaluation of an included study design | | *Does the study design meet the criteria for inclusion?*  Yes ⬜ No ⬜ 🡪**Exclude** Unclear ⬜ |  |
|  | Description in text: | | |  |
| **Participants**  (Review authors insert inclusion criteria as defined in Protocol) | Describe the participants included: | | |  |
|  | Are participants defined as a group having specific social or cultural characteristics? | Yes ⬜ No ⬜ Unclear ⬜  Details: | |  |
|  | How is the geographic boundary defined? | Details:  Specific location (e.g. state / country): | |  |
|  | *Do the participants meet the criteria for inclusion?* | Yes ⬜ No ⬜ 🡪**Exclude** Unclear ⬜ | |  |

| **Types of intervention**  (Review authors insert inclusion criteria as defined in Protocol) | Strategies included in the intervention | |  | |  |
| --- | --- | --- | --- | --- | --- |
|  | Focus of the intervention | |  | |  |
|  | *Does the intervention meet the criteria for inclusion?* | | Yes ⬜ No ⬜ 🡪**Exclude** Unclear ⬜ | |  |
| **Duration of intervention** | Start date: | Stop date: | | Intervention duration: |  |
|  | *Is the duration of intervention adequate for inclusion?* | | Yes ⬜ No ⬜ 🡪**Exclude** Unclear ⬜ | |  |
| **Types of outcome measures**  (Review authors insert inclusion criteria as defined in Protocol) | List outcomes: | |  | |  |
|  | Outcome measured at a population level or individual level? | | Details: | |  |
|  | *Do the outcome measures meet the criteria for inclusion?* | | Yes ⬜ No ⬜ 🡪**Exclude** Unclear ⬜ | |  |

**Summary of Assessment for Inclusion**

| **Include in review ⬜ Exclude from review ⬜** | |
| --- | --- |
| Independently assessed, and then compared? Yes ⬜ No ⬜ | Differences resolved Yes ⬜ No ⬜ |
| Request further details? Yes ⬜ No ⬜ | Contact details of authors: |
| **Notes:** | |

DO NOT PROCEED IF PAPER EXCLUDED FROM REVIEW

**3. Study details**

| **Study intention** | **Descriptions as stated in the report/paper** | **Page/ Para/ Figure #** |
| --- | --- | --- |
| Aim of intervention | *What was the problem that this intervention was designed to address?* |  |
| Aim of study | *What was the study designed to assess? Are these clearly stated?* |  |
| Equity pointer: Social context of the study | *e.g. was study conducted in a particular setting that might target/exclude specific population s? See also Inclusion/exclusion criteria under Methods, below.* |  |
| Start and end date of the study | *Identify which elements of planning of the intervention should be included* |  |
| Total study duration |  |  |

| **Methods** | **Descriptions as stated in the report/paper** | **Page/ Para/ Figure #** |
| --- | --- | --- |
| Method/s of recruitment of participants  *(How were potential participants approached and invited to participate? Where were participants recruited from? Does this differ from the intervention setting?)* |  |  |
| Inclusion/exclusion criteria for participation in study |  |  |
| Representativeness of sample: Are participants in the study likely to be representative of the target population? |  |  |
| Total number of intervention groups |  |  |
| Assumed risk estimate  (*e. .baseline or population risk noted in Background)* | *References:* |  |
| Sample size calculation:  What assumptions were made?  Were these assumptions appropriate? | *(Yes/No/Unclear)* |  |
| What was the unit of randomisation?  Allocation by individuals or cluster/groups |  |  |
| What was the unit of analysis?  **Is this the same as the unit of randomisation?** | *(Yes/No/Unclear)* |  |
| Statistical methods used and appropriateness of these methods | *(Check with your statistician if unsure about appropriateness)* |  |

**Results**

| **Participants**  *Include if relevant* | **Include information for each group (i.e. intervention and controls) under study** | **Page/ Para/ Figure #** |  |  |
| --- | --- | --- | --- | --- |
| 1. What percentage of selected individuals agreed to participate? |  |  |  |  |
| 1. Total number randomised (or total pop. at start of study for NRCTs) |  |  |  |  |
| 1. Number allocated to each intervention group (no. of individuals) |  |  |  |  |
| 1. For cluster trials, number of clusters, number of people per cluster |  |  |  |  |
| 1. Where there any significant baseline imbalances? | Yes ⬜ No ⬜ Unclear ⬜  Details: |  |  |  |
| 1. Number and reason for (and sociodemographic differences of) withdrawals and exclusions for each intervention group |  |  |  |  |
| 1. Were patients who entered the study adequately accounted for? |  |  |  |  |
| 1. What percentage of patients completed the study? |  |  |  |  |
| 1. What percentage of participants received the allocated intervention or exposure of interest? |  |  |  |  |
| 1. Is the analysis performed by intervention allocation status (intention to treat) rather than the actual intervention received? Have any attempts been made to impute missing data? |  |  |  |  |
| 1. Age (median, mean and range if possible) |  |  |  |  |
| 1. Sex |  |  |  |  |
| 1. Race/Ethnicity |  |  |  |  |
| 1. Principal health problem (incl. stage of illness) |  |  |  |  |
| 1. Diagnostic criteria |  |  |  |  |
| 1. Co-morbidity |  |  |  |  |
| 1. Other sociodemographics (eg. Educational level, literacy level, soci-economic status, first language. Also consider possible proxies for these e.g. low baseline nutritional status ) |  |  |  |  |
| 1. PROGRESS categories reported at baseline (indicate letters of those reported: Place of residence, race, occupation, gender, religion, education, SES, social capital) |  |  |  |  |
|  |  |  |  |  |
| **Subgroups** | *Enter a description of any participant subgroups from this paper to be analysed in the review.* |  |  |  |

**Intervention Group 1** *(copy and paste table for each Intervention group)*

| **Group name:** | *(State brief name for this intervention group.)* | **Page/ Para/ Figure #** |
| --- | --- | --- |
| Details of intervention or control condition *(Include if relevant in sufficient detail for replication)* | | |
| - Setting *eg multicentre, university teaching hospitals, rural, metropolitan, school, workplace, community, GP clinic, etc.* |  |  |
| - Theoretical basis (include key references) |  |  |
| - Content (list the strategies intended and delivered) |  |  |
| - Did the intervention include strategies to address diversity/disadvantage? | *Enter a description of any relevant strategies* |  |
| - Delivery (eg. Stages (sequential or simultaneous), timing, frequency, duration, intensity, fidelity – process indicators) |  |  |
| - Providers (who, number, education/training in intervention delivery, ethnicity etc. if potentially relevant to acceptance and uptake by participants |  |  |
| - Co-interventions |  |  |
| Duration of intervention |  |  |
| Duration of follow-up |  |  |
| Was sustainability discussed by the authors? Was is a consideration in study development? |  |  |
| Economic variables ie costs of the intervention, and changes in other (eg health care) costs as result of intervention^[[1]](#footnote-1)^♠ | Yes ⬜ 🡪**List in Outcome section if appropriate**  No ⬜ Unclear ⬜  Details: |  |
| Other economic information (from a societal, non-healthcare view – e.g. lost wages, time) | Yes ⬜  No ⬜  Details: |  |
| Resource requirements to replicate intervention (e.g. staff numbers, hours of implementation, equipment?) |  |  |
| Subgroups | *Enter a description of any intervention subgroups from this report to be analysed in the review.* |  |
| What are the moderators/mediators of changes stated in the study? |  |  |
| Do the authors describe any political or organisational context? | *List relevant dot points* |  |
| Were any partnerships referred to? | *List these as dot points* |  |
| Was a process evaluation conducted? | *What components were included in the process evaluation? (eg. dose, frequency, consistency, implemented as intended etc)* |  |
| Control/comparison (what information is provided about what the control or comparison group received?) | *Enter a description of what was provided for the control group, if applicable* |  |

**Outcomes**

*(This table is set up for 2 outcome measure to save spaces, copy and paste table as often as required*)

| **Question** | **Outcome 1** | **Page/ Para/ Figure #** | **Outcome 2** | **Page/ Para/ Figure #** |
| --- | --- | --- | --- | --- |
| Is there an analytic framework applied (e.g. logic model, conceptual framework)? |  |  |  |  |
| Outcome definition (with diagnostic criteria if relevant) |  |  |  |  |
| Type of outcome: Is this a modifiable variable (Community level, neighbourhood level, individual level) or desired health outcome |  |  |  |  |
| Time points measured |  |  |  |  |
| Time points reported |  |  |  |  |
| Is there adequate latency for the outcome to be observed? |  |  |  |  |
| Is the measure repeated on the same individuals or redrawn from the population / community for each time point? |  |  |  |  |
| Unit of measurement (if relevant) |  |  |  |  |
| For scales – upper and lower limits and indicate whether high or low score is good |  |  |  |  |
| How is the measure applied? Telephone survey, mail survey, in person by trained assessor, routinely collected data, other |  |  |  |  |
| How is the outcome reported? Self or study assessor |  |  |  |  |
| Is this outcome/tool validated? |  |  |  |  |
| …And has it been used as validated? |  |  |  |  |
| Is it a reliable outcome measure? |  |  |  |  |
| Is there adequate power for this outcome? |  |  |  |  |
|  |  |  |  |  |
| Were PROGRESS categories analysed by outcome? Indicate the letters of those that outcomes were analysed by (place of residence, race, occupation, gender, religion, education, SES, social capital) |  |  |  |  |

**Results**

*Copy and paste the appropriate table for each outcome and subgroup at each timepoint, including baseline*

**For RCT/CCT**

**Dichotomous outcome** page/para/fig

| Comparison |  | | | |  |
| --- | --- | --- | --- | --- | --- |
| Outcome |  | | | |  |
| Subgroup |  | | | |  |
| Timepoint |  | | | |  |
| **Results** | **Intervention** | | **Comparison** | |  |
|  | Events | No. participants | Events | No. participants |  |
|  |  |  |  |  |  |
| No. of missing participants and reasons |  | |  | |  |
| Any other results reported |  | | | |  |
| Reanalysis required? (specify -  (e.g. correlation adjustment) |  | | | |  |
| Reanalysis possible? | *yes/no/unclear* | | | |  |
| Reanalysed results |  | | | |  |

**For RCT/CCT**

**Continuous outcome** page/para/fig

| Comparison |  | | | | | |  |
| --- | --- | --- | --- | --- | --- | --- | --- |
| Outcome |  | | | | | |  |
| Subgroup |  | | | | | |  |
| Timepoint |  | | | | | |  |
| Post-intervention or change from baseline? |  | | | | | |  |
| **Results** | **Intervention** | | | **Comparison** | | |  |
|  | Mean | SD (or other variance) | No. participants | Mean | SD (or other variance) | No. participants |  |
|  |  |  |  |  |  |  |  |
| No. missing participants and reasons |  | | |  | | |  |
| Any other results reported |  | | | | | |  |
| Reanalysis required? (specify) |  | | | | | |  |
| Reanalysis possible? | *yes/no/unclear* | | | | | |  |
| Reanalysed results |  | | | | | |  |

**For RCT/CCT**

**Generic inverse variance method**

Page/para/figure

| Comparison |  | | | |  |
| --- | --- | --- | --- | --- | --- |
| Outcome |  | | | |  |
| Subgroup |  | | | |  |
| Timepoint |  | | | |  |
| Results | Effect estimate | SE (or other variance) | Intervention no. | Control no. |  |
|  |  |  |  |  |  |
| No. missing participants and reasons |  | | | |  |
| Any other results reported |  | | | |  |
| Reanalysis required? (specify) |  | | | |  |
| Reanalysis possible? | *yes/no/unclear* | | | |  |
| Reanalysed results |  | | | |  |

**For CBA**

Page/para/fig

| Comparison |  | |  |
| --- | --- | --- | --- |
| Assignment | How were control and treatment groups selected?? Is there likely to be an effect if these were the opposite way? | |  |
|  | Contemporaneous data collection? | |  |
| Outcome |  | |  |
| Subgroup |  | |  |
| Timepoint |  | |  |
| Post-intervention or change from baseline? |  | |  |
|  | **Intervention** | **Comparison** |  |
| No. participants  measured |  |  |  |
| No. missing participants and reasons |  |  |  |
| Baseline result (with variance measure) |  |  |  |
| Post-intervention results (with variance measure) |  |  |  |
| Change (Post – baseline) (with variance measure) |  |  |  |
| Difference in change (intervention – control) (with variance measure) |  | |  |
| Any other results reported |  | |  |
| Reanalysis required? (specify) |  | |  |
| Reanalysis possible? | *yes/no/unclear* | |  |
| Reanalysed results |  | |  |

**For ITS**

**Generic inverse variance method** Page/para/fig

| Comparison |  | | | | |  |
| --- | --- | --- | --- | --- | --- | --- |
| Outcome |  | | | | |  |
| Subgroup |  | | | | |  |
| Length of timepoints measured |  | | | | |  |
| Snapshot or interval measured |  | | | | |  |
| No. participants measured |  | | | | |  |
| No. missing participants and reasons |  | | | | |  |
|  | Pre-intervention | | | Post-intervention | |  |
| No. of timepoints measured |  | | |  | |  |
| Mean value (with variance measure) |  | | |  | |  |
| Difference in means (post – pre) |  | | | | |  |
| Percent relative change |  | | | | |  |
| Result reported by authors (with variance measure) |  | | | | |  |
| Reanalysis required? (specify) |  | | | | |  |
| Reanalysis possible? | *yes/no/unclear* | | | | |  |
| Individual time point results |  | | | | |  |
| Read from figure? | *yes/no* | | | | |  |
| Reanalysed results | Change in level | SE | Change in slope | | SE |  |
|  |  |  |  | |  |  |

**Other relevant information**

| Were outcomes relating to harms/unintended effects of the intervention described? Include any data for these in the outcomes tables above |  |  |  |
| --- | --- | --- | --- |
| Potential for author conflict *ie. evidence that author or data collectors would benefit if results favoured the intervention under study or the control* |  |  |  |
| Key conclusions of the study authors |  |  |  |
| Could the inclusion of this study potentially bias the generalisability of the review? Equity pointer: Remember to consider whether disadvantaged populations may have been excluded from the study. |  | | |
| Is there potential for differences in relative effects between advantaged and disadvantaged populations? (e.g. are children from lower income families less likely to wear bicycle helmets) |  | | |
| Are interventions likely to be aimed at the disadvantaged? (e.g. school meals aimed at poor children). |  | | |
| Issues affecting directness  (*Note any aspects of population, intervention, etc. that affect this study’s direct applicability to the review question)* |  | | |
| References to other relevant studies |  | | |
| Additional notes by review authors |  | | |
| Correspondence required for further study information (from whom, what and when) |  | | |

**Risk of bias assessment**

Please refer to Chapter 8 - *Table 8.5.c: Criteria for judging risk of bias in the ‘Risk of bias’ assessment tool and to the Cochrane EPOC Group’s guidance for assessing* **Risk of bias for studies with a separate control group (RCTs, CCTs, CBAs) and Risk of bias for interrupted time series studies** (Appendix 3) for additional guidance for scoring Yes/No/Unclear. Note that the table below includes items from both EPOC tools. The ITS tool has been incorporated into the bottom of the table and all items for ITS studies are denoted by ITS preceding the risk of bias question.

| **Domain** | **Review authors’ judgement*** | **Description** | **Page/ Para/ Figure #** |
| --- | --- | --- | --- |
| **Was the allocation sequence adequately generated?** | **Yes / No / Unclear** | ***Describe the method used to generate the allocation sequence in sufficient detail to allow an assessment of whether it should produce comparable groups.*** |  |
| **Was allocation adequately concealed?** | **Yes / No / Unclear** | ***Describe the method used to conceal the allocation sequence in sufficient detail to determine whether intervention allocations could have been foreseen in advance of, or during, enrolment.*** |  |
| **Were baseline outcome measurements similar?** | **Yes/No/Unclear** | ***Note whether baseline outcome measurements were reported and whether there were any important differences between groups. If there were important differences between groups, note whether appropriate adjusted analysis was performed to account for this.*** |  |
| **Were baseline characteristics similar?** | **Yes/No/Unclear** | ***Note whether baseline characteristics were reported and whether there were any important differences between groups.*** |  |
| **Were incomplete outcome data adequately addressed?**  ***Assessments should be made for each main outcome (or class of outcomes).*** | **Yes / No / Unclear** | ***Describe the completeness of outcome data for each main outcome, including attrition and exclusions from the analysis. State whether attrition and exclusions were reported, the numbers in each intervention group (compared with total randomized participants), reasons for attrition/exclusions where reported, and any re-inclusions in analyses performed by the review authors.*** |  |
| **Was knowledge of the allocated intervention adequately prevented during the study?**  ***Separate assessments should be made for relevant groups of people involved in the study i.e participants, outcome assessors, investigators, data assessors etc*** | **Yes / No / Unclear** | ***Describe all measures used, if any, to blind study participants and personnel from knowledge of which intervention a participant received. Provide any information relating to whether the intended blinding was effective, or whether blinding was appropriate.***   1. Participants – yes, no, unclear *[record supporting statement from study].* 2. Investigators – yes, no, unclear *[record supporting statement from study].* 3. Outcomes assessors – yes, no, unclear *[record supporting statement from study].*   Data assessors – yes, no, unclear *[record supporting statement from study].* |  |
| **Was the study adequately protected against contamination?** | **Yes/No/Unclear** | ***State whether and how the possibility of contamination was minimised by the study design/implementation.*** |  |
| **Are reports of the study free of suggestion of selective outcome reporting?**  ***Assessments should be made for each main outcome (or class of outcomes).*** | **Yes / No / Unclear** | ***State how the possibility of selective outcome reporting was examined by the review authors, and what was found.*** |  |
| **Other sources of bias** | **Yes / No / Unclear** | ***State any important concerns about bias not addressed in the other domains in the tool.*** |  |
| **ITS: Was the intervention independent of other changes?** | **Yes/No/Unclear** | ***Describe whether or not the intervention occurred independently of other changes over time and whether or not the outcomes may have been influenced by other confounding variables/historic events during the study period.*** |  |
| **ITS: Was the shape of the intervention effect pre-specified?** | **Yes/No/Unclear** | ***State whether or not the point of analysis was the point of intervention. If not, describe whether a rationale for the shape of the intervention effect was given by the study authors.*** |  |
| **ITS: Was the intervention unlikely to affect data collection?** | **Yes/No/Unclear** | ***Describe whether or not the intervention was likely to affect data collection and what the potential impact might have been.*** |  |
| **ITS: Was knowledge of the allocated interventions adequately prevented during the study?**  ***Separate assessments should be made for relevant groups of people involved in the study i.e participants, outcome assessors, investigators, data assessors etc*** | **Yes/No/Unclear** | ***Describe all measures used, if any, to blind study participants and personnel from knowledge of which intervention a participant received. Provide any information relating to whether the intended blinding was effective, or whether blinding was appropriate.***   1. Participants – yes, no, unclear *[record supporting statement from study].* 2. Investigators – yes, no, unclear *[record supporting statement from study].* 3. Outcomes assessors – yes, no, unclear *[record supporting statement from study].*   Data assessors – yes, no, unclear *[record supporting statement from study].* |  |
| **ITS: Was incomplete outcome data adequately addressed?**  ***Assessments should be made for each main outcome (or class of outcomes).*** | **Yes/No/Unclear** | ***Describe the completeness of outcome data for each main outcome, including attrition and exclusions from the analysis. State whether attrition and exclusions were reported, the numbers in each intervention group (compared with total randomized participants), reasons for attrition/exclusions where reported, and any re-inclusions in analyses performed by the review authors.*** |  |
| **ITS: Was the study free from selective reporting?** | **Yes/No/Unclear** | ***State how the possibility of selective outcome reporting was examined by the review authors, and what was found.*** |  |
| **ITS: Was the study free from other risks of bias?** | **Yes/No/Unclear** | ***State any important concerns about bias not addressed in the other domains in the tool.*** |  |

* Note: For each section above ‘Yes’ indicates a ‘low risk of bias’; ‘No’ indicates a ‘high risk of bias’; ‘Unclear’ indicates an ‘uncertain risk of bias’. When entering the data into RevMan, the options to choose from will be ‘Low’, ‘High’ and ‘Unclear’

**Results**

Comparison:

Outcome:

Subcategory:

| **Treatment group:** | | **Control group:** | |
| --- | --- | --- | --- |
| Observed (n) | total (N) | observed (n) | total (N) |
|  |  |  |  |

|  | **Treatment group:** | **Control group:** |
| --- | --- | --- |
| Total randomised |  |  |
| excluded* |  |  |
| Observed |  |  |
| lost to follow up* |  |  |

*Reasons for loss/exclusion:

Subcategory:

| **Treatment group:** | | **Control group:** | |
| --- | --- | --- | --- |
| Observed (n) | total (N) | observed (n) | total (N) |
|  |  |  |  |

|  | **Treatment group:** | **Control group:** |
| --- | --- | --- |
| Total randomised |  |  |
| excluded* |  |  |
| Observed |  |  |
| lost to follow up* |  |  |

*Reasons for loss/exclusion

1. ♠ Costs associated with the intervention can be linked with provider or participant outcomes in an economic evaluation (depends on the type of economic evaluation) [↑](#footnote-ref-1)
